# Supplementary figures and images for: Bats generate lower affinity but higher diversity antibody responses than those of mice, but pathogen-binding capacity increases if protein is restricted in their diet
Source: PLoS Biol. 2024 Sep 24;22(9):e3002800. doi: 10.1371/journal.pbio.3002800 (PMC11421821; doi:10.1371/journal.pbio.3002800)

| *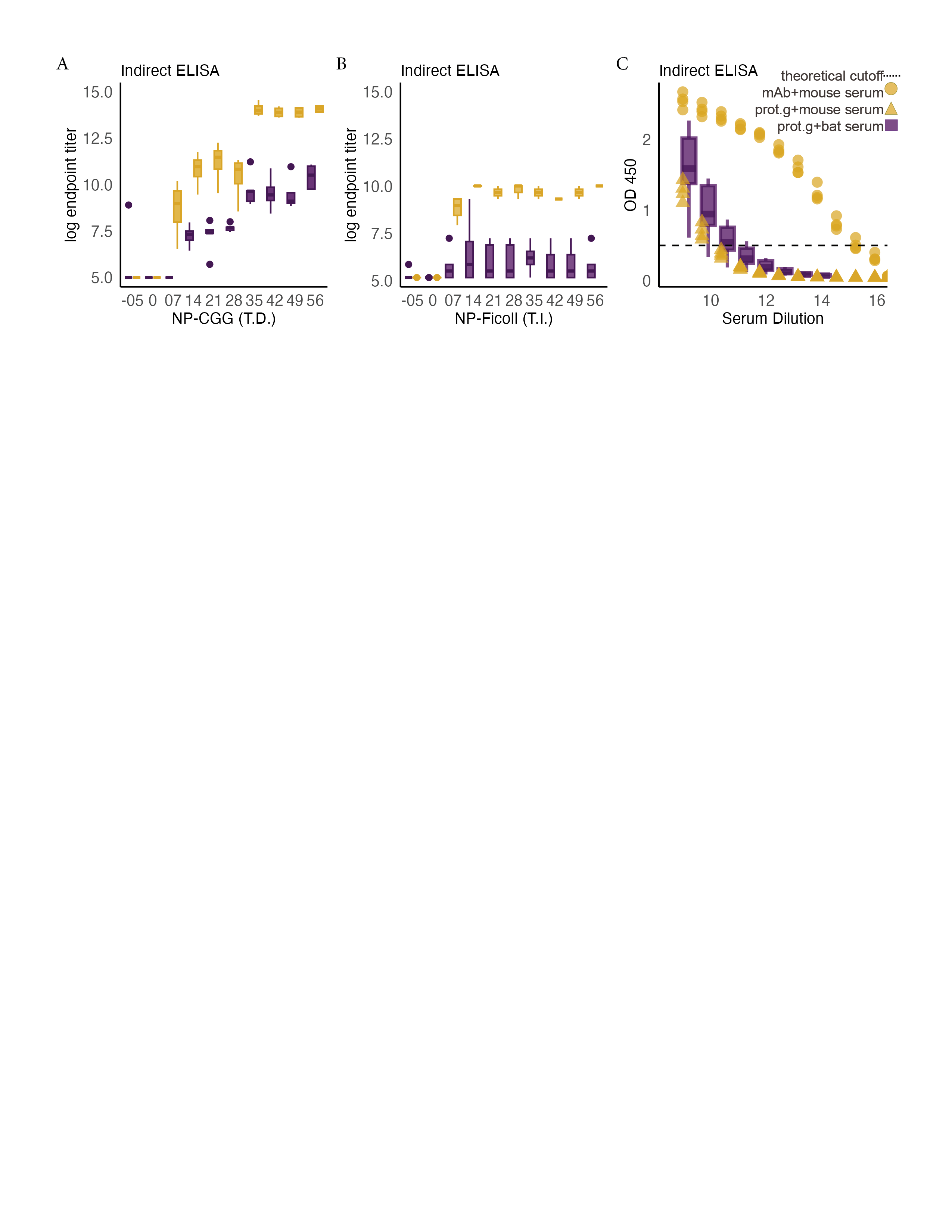* |
| --- |
| **Supplemental Figure 1** |

Supplement: S1 Fig — (A) Indirect ELISA endpoint titers from bats and mice immunized and boosted with NP-CGG (T-dependent). (B) Indirect ELISA endpoint titers from bats and mice immunized and boosted with NP-Ficoll (T-independent). (C) Indirect ELISA dilution curve from bats and mice immunized and boosted with NP-CGG. Serum is from day 56. Bat serum IgG was measured using protein-G as the secondary reagent. Mouse serum IgG was measured with either protein-G or a monoclonal antibody specific to mouse IgG as the secondary reagent. The dashed line at 0.5 indicates a theoretical OD450 cutoff to establish an endpoint titer. Serum dilution is shown on a natural scale. Boxplots show the 25%, 50%, 75% percentiles, lines indicate the smallest and largest values within 1.5 times the interquartile range, dots indicate values beyond that. When an endpoint titer could not be estimated, the value was set to that of the pre-immunization serum. P values derived from F statistic. Significance codes: “***”P < 0.001, “**”P < 0.01, “*”P < 0.05. All code and data to recreate figures can be found at https://zenodo.org/records/12825679. (DOCX) [file pbio.3002800.s001.docx]

| 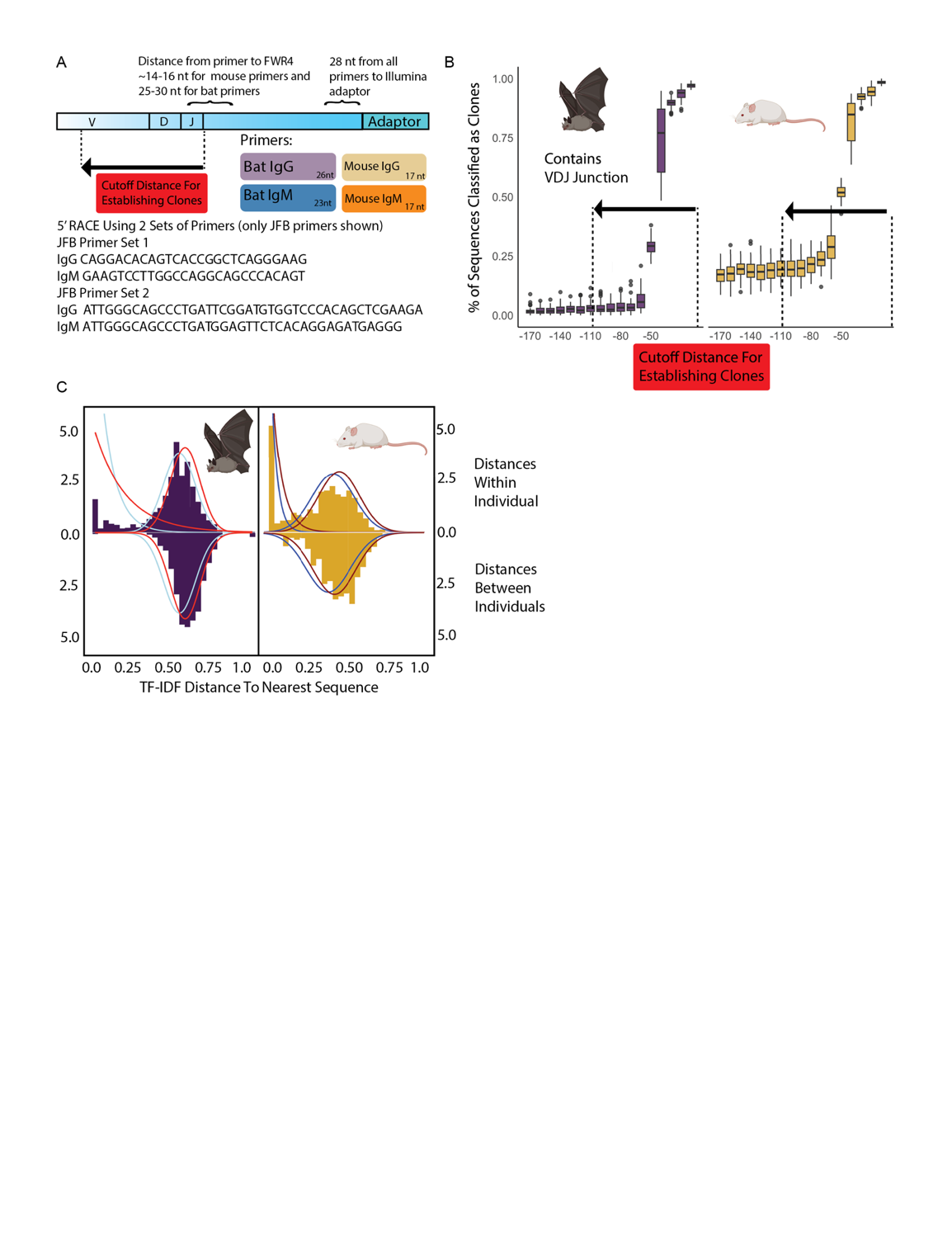 |
| --- |
| **Supplemental Figure 2** |

Supplement: S2 Fig — (A) Graphical representation of BCR mRNA. Shown is the VDJ region, where much of the BCR diversity occurs. Also shown is the Illumina adaptor and the location where the species and Ig class-specific primers bound. The “Cutoff Distance for Establishing Clones” is a graphical representation of how many nucleotides were used to build our diversity models. (B) We assessed a range of cutoff values to build our diversity models. Before modeling diversity, we needed to classify mRNA BCR transcripts into clones. Without an annotated V, D, and J germline for Artibeus bats, we classified BCR transcripts into clones using the TF-IDF distance metric. We optimized the number of nucleotides to include when calculating the TF-IDF metric. We refer to the number of nucleotides as the “Cutoff Distance for Establishing Clones.” For the optimization, we repeatedly subsampled sequences from our full BCR sequence library. For each sample, we trimmed the 5′ end of the sequence. After trimming the subsample, we clustered sequences into clones and calculated the percentage of sequences that classified as clones. When the “Cutoff Distance for Establishing Clones” was <50 nucleotides, the BCR sequence primarily consisted of the constant region and some of the FWR4 region. Since there is no variability in the constant region of the BCR sequence, almost all sequences were classified as a single clone. As the cutoff distance approached 110 nucleotides, the trimmed sequences encapsulated to VDJ region, which is the most variable region of the BCR sequence. (C) Histogram of the TF-IDF distance metric used to cluster BCR sequences. TF-IDF was used to compare sequence similarity. To establish a TF-IDF cutoff, we used a Bayesian mixture models to identify the TF-IDF cutoff that could cluster a group of sequences as a set of “clones.” The posteriors of a Bayesian hierarchical mixture model are shown in red and the results from a nonhierarchical mixture model are shown in blue. Sequences shown here are [file pbio.3002800.s002.docx]

| *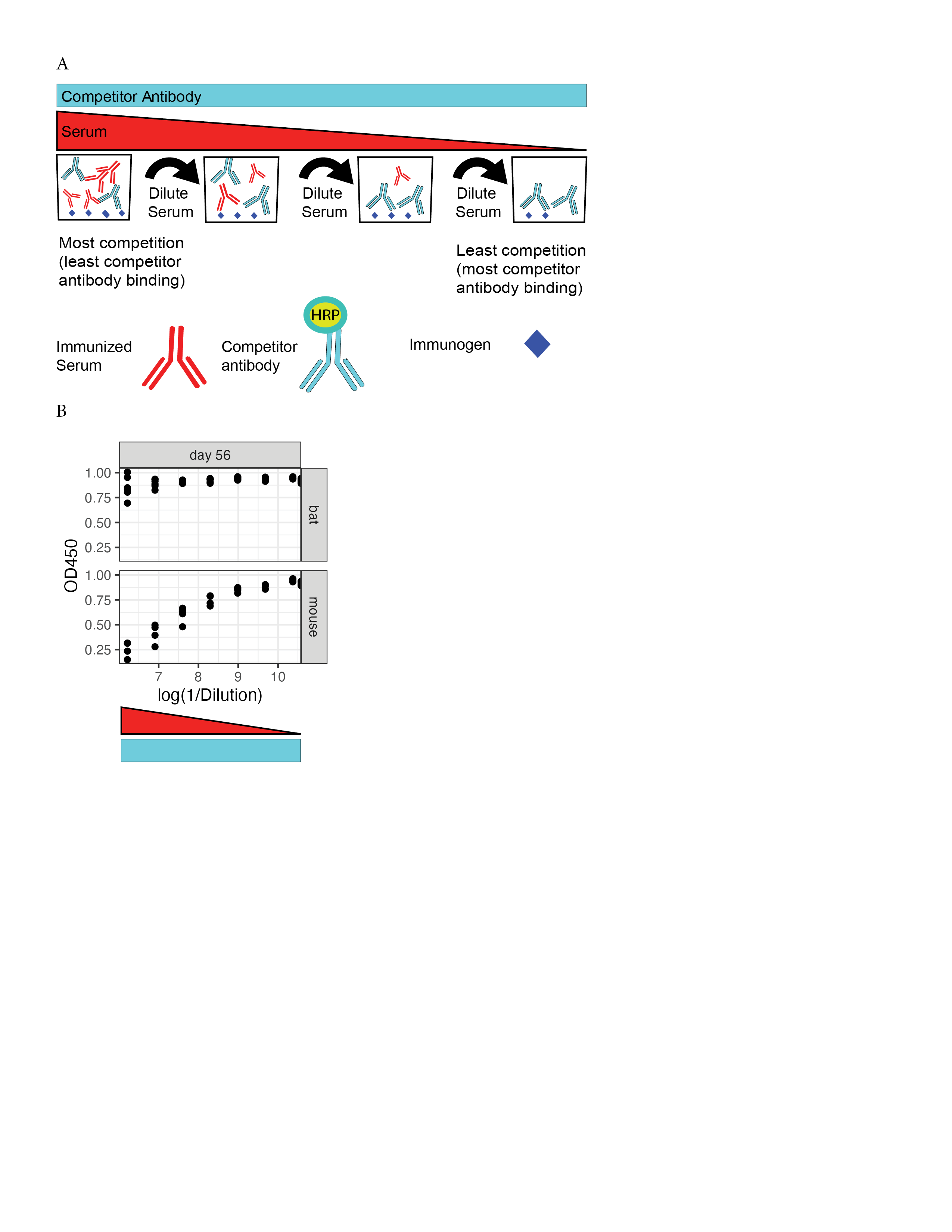* |
| --- |
| **Supplemental Figure 3** |

Supplement: S3 Fig — (A) An in-depth visual of the competition ELISA. Data shown comes from animals immunized with NP-CGG. The serum is from day 56. (B) As immunized animal serum is diluted out the OD450 increases. OD450 is indicating the amount of tagged competitor antibody that is binding. This approach is an attempt to remove the bias introduced by species-specific reagents and the bias introduced by protein-G’s different affinities for different species. All code and data to recreate figures can be found at https://zenodo.org/records/12825679. (DOCX) [file pbio.3002800.s003.docx]

| *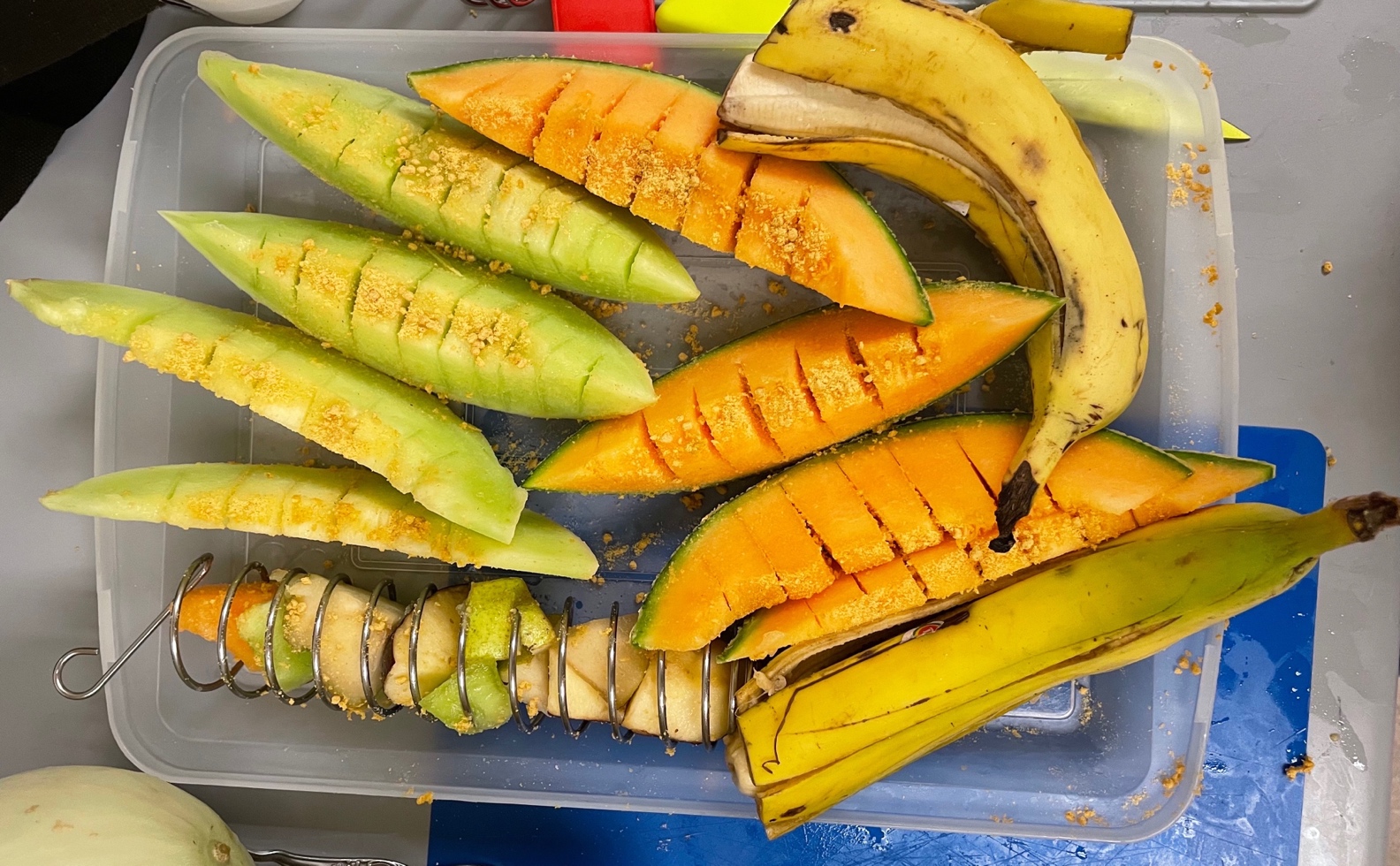* |
| --- |
| **Supplemental Figure 4** |

Supplement: S4 Fig — The fruits in this photo are coated in the ground Mazuri Softbill Protein. (DOCX) [file pbio.3002800.s004.docx]
