# Supplementary material for: Bats generate lower affinity but higher diversity antibody responses than those of mice, but pathogen-binding capacity increases if protein is restricted in their diet
Source: PLoS Biol. 2024 Sep 24;22(9):e3002800. doi: 10.1371/journal.pbio.3002800 (PMC11421821; doi:10.1371/journal.pbio.3002800)
Supplement: S1 Table — (DOCX) [file pbio.3002800.s005.docx]

| ** |
| --- |
| ***Supplemental Table 1*** |
