# Supplementary material for: Bats generate lower affinity but higher diversity antibody responses than those of mice, but pathogen-binding capacity increases if protein is restricted in their diet
Source: PLoS Biol. 2024 Sep 24;22(9):e3002800. doi: 10.1371/journal.pbio.3002800 (PMC11421821; doi:10.1371/journal.pbio.3002800)
Supplement: S1 Methods — (DOCX) [file pbio.3002800.s006.docx]

**Supplemental Methods**

Indirect ELISA Assays

Antibody titers were assessed for all blood samples from experiments one, two, and four. To determine endpoint titers, we coated a 96-well ELISA plate with 100 µL of antigen: KLH (Sigma Aldrich SRP6195), NP-CGG (Santa Cruz Biotech sc-396209), NP-Ficoll (Santa Cruz Biotech sc-396292) or recombinant H18N11 hemagglutinin (Sinobiological Influenza A-like H18 Hemagglutinin Cat: 40324-V08) at a concentration of 1 µg/mL, diluted in sodium bicarbonate buffer (2.93 g NaHCO_3_ & 1.59 g Na_2_CO_3_ L-1, pH 9.6), followed by a two-hour room temperature (RT) incubation. We washed the plates three times with PBST, blocked with 200 µL of NAP buffer (one part NAP and two parts PBST) for one hour RT. We serially diluted the serum into NAP buffer, added 100 µL to each well, and incubated the plate for two hours RT. We washed the plates three times in PBST and probed using protein G:HRP, goat anti-IgG-Heavy chain HRP antibody (Southern Biotech Cat. No.: 1031-05), or our own monoclonal antibody F4. Endpoint titers were the reciprocal of the estimated dilution that generated a value three standard deviations above the unimmunized serum control. If serum antibodies were not detected, either because the samples were taken prior to immunization or the animal had not yet developed a detectable antibody response, we reported the titer as the strongest dilution tested. If the weakest dilution tested still had an OD450 above background the endpoint titer was set to weakest dilution tested.

Competition ELISAs

We designed a competition ELISA to compare the antibody responses between JFB and mice. This competition ELISA was designed to minimize bias introduced by secondary reagents for mouse and bat antibodies (S1C Fig). Protein G, a commonly used secondary reagent for detecting bat antibodies, possesses species-specific Ig affinities, biasing results even within a genus^77^. For the competition assay, we diluted immunized animal serum from a 1:100 dilution to a 1:1.5 x 10^5^ dilution. This dilution series of serum was combined with a single dilution of rabbit anti-NP monoclonal antibody B1-8 (Abcam ab289966). The dilution of B1-8 was 2 µg/µL for NP-CGG or 4 µg/µL for NP-Ficoll tests. ELISA plates were coated with NP-CGG or NP-Ficoll in sodium bicarbonate at a concentration of 0.5 µg/mL. The serum and B1-8 mAb mixture was added to the plates and incubated for two hours at room temperature. For the NP-CGG assays, detection was done using an anti-rabbit monoclonal antibody. For the NP-Ficoll assays, detection was done by biotinylating (Abcam ab201795) the B1-8 mAb and using avidin-HRP at a 1:5000 dilution as our detection reagent. The endpoint titer was the estimated serum concentration where no displacement of the B1-8 competitor was observed.

B Cell Receptor (BCR) Sequencing

We sequenced the mRNA from secondary lymphoid tissue of JFB and mice. Briefly, we harvested mRNA from spleen tissue or MLN harvested at necropsy of animals at terminal time points. Spleens and MLNs were flash-frozen in liquid nitrogen upon collection. Tissues were lyophilized and bead beaten in Trizol. RNA was enriched using the Invitrogen Phasemaker kit (Invitrogen A33254). The RNA was subject to a DNAse treatment (Life Technologies AM1907).

For BCR library preparation, we followed previous protocols, but used JFB IgM and IgG primers for bat tissue (S2A Fig) ^78^. We prepared sequence libraries with the NEBNext Ultra# II DNA Library Prep with Sample Purification Beads (cat# E7103S) and NEBNext Multiplex Oligos for Illumina (Index Primers Set 1, cat# E7335S). We cleaned the samples using Sample Purification Beads and enriched libraries for 600-700 BP amplicons according to the manufacturer instructions. We checked the size and quality of sequences on a fragment analyzer to ensure that libraries had a single band between 650-750 BP in length. Only samples from the same Illumina run were analyzed together. All libraries were run using the MiSeq 2x300 chemistry Illumina platforms.

Bioinformatics Steps

We processed, filtered, and analyzed the sequencing BCR reads using pRESTO and previously described germline free identification methods^79,80^. We removed sequences with average Fastq quality score below 30. Consensus sequences were built from shared unique molecular identifiers (UMI). Sequences with low quality ends (<20) were trimmed. Sequences underwent sequential assembly. Both mouse and JFB reads which failed the R1 R2 alignment were aligned to mouse germline sequences and placeholder nucleotides “N” were filled into the R1-R2 gap. Sequences were only included in downstream analysis that IgBLAST classified as a functional BCR sequence (IgBlast version 1.18.0). For IgBlast, initially we used mouse germline V,D,&J sequences for both mouse and JFB sequences; however, no germline assignments were used in downstream analyses as we found a mismatch between the sequence and germline species introduced unpredictable biases and errors. Mouse germline sequences were obtained from the ﻿the international ImMunoGeneTics/GENE database (IMGT/GENE-DB) ^81^ on 2022-03-20. Finally, the 3’ C region of sequences were trimmed.

Sequence diversity was assessed using a germline free identification method as previously described ^80^. The two outputs of this germline-free identification method were the TF-IDF distance metric within and between individuals. TF-IDF (term frequency, inverse document frequency) provides a germline free measurement of BCR sequence similarity, as described previously^80^. We tested a range of cut off distances to establish BCR clones (S2B Fig). We refer to this cutoff distance as the “Cutoff Distance for Establishing Clones” in S2 Fig. This value represents the number of nucleotides from the 3’ end of the BCR sequence used in the germline free identification. We tested the impact of the cutoff distance (L) from 10 nucleotides to 200 nucleotides. We visually assessed the value where the within-individual TF-IDF distance metric stabilized and where the between-individuals TF-IDF distance metric approached zero for both mouse and JFB sequences (S2B Fig). Amplicons shorter than the cutoff distance (L) were filtered out of subsequent analyses. We built a hierarchical Bayesian mixture model using Rstan 2.21.8 to bin sequences by the TF-IDF distance into clones (S2C Fig) ^82^. After binning sequences, we assessed the alpha diversity of BCR sequences cluster (“clones”) using Hill Diversity Curves^41,83,84^. Statistical significance tests of the Hill Diversity Curves were done at the species level as described previously^83^.

Pseudotyped Virus Neutralization Assay

Sera from Nipah-riVSV virion-inoculated bats were diluted from a 1:10 to a 1:3x10^4^ dilution. Diluted serum was combined with a 1:10^4^ dilution NiV F/G pseudotyped VSV particles, which encoded a luciferase marker gene. Pseudotyped VSV virions were treated with equal amounts of each serum dilution and incubated for one hour at 37^o^C. HEK239T cells were seeded 24 hours prior to achieve 40% confluency at the time of infection and infected with the serum-treated virus. Neutralization of the pseudotyped VSV was measured using the luciferase assay after 24 hours. Statistical significance was assessed by comparing to the neutralization output to day zero values. A Bonferroni multiple test correction was used.

Monoclonal Antibody Production

Hybridoma derived monoclonal antibodies were generated using BALB/c splenocytes and SP2/0 myeloma cells. Previously, BALB/c mice were immunized and boosted i.p. with S-300 size fractionated *Pteropus* serum enriched for IgM and IgA antibodies and Hunters Titermax Gold adjuvant. Spleens from these mice were harvested four days after the final boost and fused with SP2/0 myeloma cells to create hybridomas, per standard protocols. The hybridoma derived antibodies were screened for cross-reactivity with *Pteropus* and JFB serum via ELISA. Positive hybridomas were subcloned two times and then screened via western blot.
