# Supplementary material for: Bats generate lower affinity but higher diversity antibody responses than those of mice, but pathogen-binding capacity increases if protein is restricted in their diet
Source: PLoS Biol. 2024 Sep 24;22(9):e3002800. doi: 10.1371/journal.pbio.3002800 (PMC11421821; doi:10.1371/journal.pbio.3002800)
Supplement: S1 Raw Images — (PDF) [file pbio.3002800.s007.pdf]

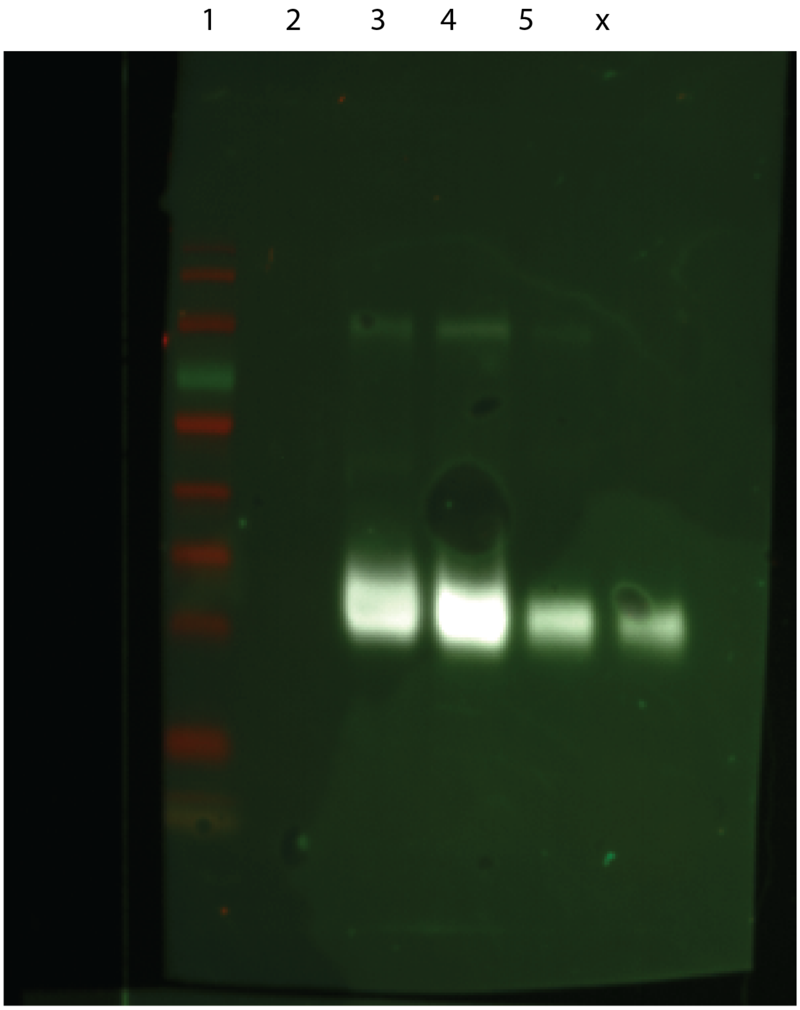

1: Ladder

2: PBS

3. Bat serum enriched for IgM

4. Bat serum enriched for IgA

5. Bat serum enriched for IgG

x. Non-enriched serum

Blot is from a culture supernatant from a hybridoma we made ("F4"). The hybridoma derived monoclonal antibodies were generated using BALB/c splenocytes and SP2/0 myeloma cells. Previously, BALB/c mice were immunized and boosted i.p. with S-300 size fractionated bat serum enriched for IgM and IgA antibodies and Hunters Titermax Gold adjuvant. Spleens from these mice were harvested four days after the final boost and fused with SP2/0 myeloma cells to create hybridomas, per standard protocols. Positive hybridomas were subcloned two times and then screened via western blot.

The western blot is bat serum that has been enriched via S300 bead size chromotography for specific antibodies.

The image was taken with a model FluorChem R from Protein Simple.
