# Supplementary material for: Bats generate lower affinity but higher diversity antibody responses than those of mice, but pathogen-binding capacity increases if protein is restricted in their diet
Source: PLoS Biol. 2024 Sep 24;22(9):e3002800. doi: 10.1371/journal.pbio.3002800 (PMC11421821; doi:10.1371/journal.pbio.3002800)
Supplement: Attachment — Submitted filename: Crowley_et_al_reviewer_responses_2023_12_27.pdf [file pbio.3002800.s008.pdf]

Second round of reviewer comments:

Reviewer #1:

Comments:
In this revised study by Crowley, they have improved the presentation of the data which greatly improves the readability of the paper.

There are still aspects that need to be addressed to make the findings more scientifically robust:

1) Quantification required in the dietary change study: this is an important aspect of the paper in the second part of the study. But, the change is not quantified at all - usually some quantification of the macro and micro nutrients is needed for dietary studies, not a rough statement like "protein powder was included with fruits". Also, there was no quantification for intake per bat during the study.

> R1.b: We agree that it is important to state the quantities. We now specify the quantity of protein powder added to the fruit and clarify that each group received the same amount of fruit. We also include a photograph of a representative meal. The bats were provided food ad libitum (as detailed in the text).

2) Lack of mechanistic data: drop of neutralising antibody titers with a protein rich diet is an important and intriguing observation, but no attempt/data was given in understanding the mechanism of this highly central observation.

> R2.b: We included B cell receptor sequencing data that offers a mechanistic insight into this observation. The changes in sequence diversity suggest potential alterations in the B cell repertoire following dietary changes. We also noted shifts in antibody mRNA location, as we observed a shift in where antibody mRNA sequences were found, moving from the spleen to the lymph node. This may indicate changes in B cell activation and differentiation.

Reviewer #3:

Comments:
This is an interesting study representing a considerable amount of work. However, key methodological details are missing which prevent me from fully evaluating the data analysis performed.

The conclusions could be strengthened considerably by better placing results within the context of previous work. In particular, while this study examines just one bat species, most of the text assumes that the results apply to all or most bats despite the incredible diversity of this group. For the antibody affinity results, this assumption makes sense in light of previous studies, but that this is the case is never explained to readers not familiar with these studies. It would help to explicitly state the species assessed in previous work (e.g., from line 45 onward), and ideally to also add discussion text on just how broadly (in terms of antigens, bat taxonomy, and bat diet) comparable results have now been found. For the other results (i.e., diversity, link to diet, etc.), the wording needs to be more careful - this is the novel part of this study, so how broadly this applies to other species / clades remains to be seen. More generally, I think the text and particularly the significance statement and figures should make clear what species is being discussed, rather than referring to "bats" when just one species is used. Generalisations can then be made in the abstract and discussion, where there is sufficient space to provide the needed context.

> R3.b: In mouse studies, the strain (e.g., C57BL/6 and BALB/c) is sometimes specified and then "mouse" is subsequently used to improve readability. Using ‘bats’ reduces the need for acronyms (e.g., JFB for

Jamaican fruit bats). However, we agree that our findings should not be generalized across all bat species, and have addressed this by adding the following sentences to the discussion (on lines 218 to 220): *"Furthermore, while the JFB are a widely used model species for bat research, they are one of more than 1400 species of bats [50], and it is important to not assume these traits are conserved across all bat species."* We also changed the first sentence of the discussion (lines 174-175) to remind the reader that these results are specifically referring to the Jamaican fruit bat:
"Our results suggest the Jamaican fruit bats' antibody responses are indeed weaker compared to mice, however an improved antibody response can be observed if one removes protein from their diet."

However, we do note that this phenomenon has now been observed in two disparate bat families: by correlative field studies in Pteropodid bats, and by our experimental studies in Phyllostomid bats.

Care is also needed to avoid implying conclusions not supported by the presented results. This applies particularly to the impact of protein restriction. Since no data on caloric intake or weight changes are presented (see comment for line 270 below), we cannot be sure that removing protein had a direct causal impact on antibody binding affinity. All we know is that altering diet in some way correlates with affinity - a significant result, but one that requires further investigation to find the mechanism. Thus I think consistent use of the phrase "diet manipulation" (or some other qualification) would be better than causal statements like "binding capacity can be improved by removing protein from their diet" (line 35), "an improved antibody response can be observed if one removes protein from their diet" (line 176), etc.

R4.b: Our study provides one of very few attempts at establishing causality related to bat viral ecology. This is a new field, and we are forging our way forward in new territory. We established a bat colony, developed new methods to sequence the B cell receptor of Jamaican fruit bats, generated new monoclonal antibodies to measure the immune response of bats, and optimized new laboratory methods to enable accurate comparisons of bats with other species. We implemented two unique experimental models which enabled us to manipulate bats' diets to examine ecological phenomena that have been linked to spillover of World Health Organization priority pathogens from bats (Eby et al. 2023, Nature). Our work provides a significant advance in the field and paves the way for more work to understand the unique bat immune response.

At some spatial and temporal scale, most observations in science are correlational. The field involved with trying to understand drivers of bat viral spillover has been especially so, probably because bats and their viruses are situated in complex ecological systems that span spatial and organizational scales from proteins to landscapes, and bats have rarely been taken into the laboratory. Our study is one of very few studies to take the step to replicate phenomena observed in the field and manipulate the putative causal factors in the lab. We randomized bats to dietary groups to minimize confounding, we repeated our first experiment, taking a year to increase the rigor to evaluate the pattern of antibody titer related to diet. Although we cannot meet the standards of well-characterize mouse systems where many reagents have been available for decades, we make a first major step towards understanding the role of immunity in driving emerging infectious diseases from bats, a non-model system for which almost no reagents or methods have been developed.

Major comments:
Line 201 - 203: "Our findings suggest the food bats consume directly impacts their antibody response and offer a mechanistic connection between the quality of antibody responses in bats and ecological conditions." What is this mechanism? So far only associations/correlations have been shown, but it's unclear how less protein in the diet causes changes in BCR sequence diversity and antibody affinity. Perhaps a more detailed explanation (aimed at general PNAS readers, not just immunologists) would help.

R5.b: The paragraph referenced above presents correlational data from field studies. Our present work leverages controlled experimental models (described in R4.b) to establish a causal link between dietary factors and antibody responses in bats. This mechanistic understanding provides a direct explanation for the previously observed associations between bat antibody responses and ecological conditions in field studies. We have changed the wording to clarify this.

Line 265: "The pregnant bat was not included in analyses unless stated otherwise." The pregnant bat is not mentioned elsewhere at all.

R6.b: We did not mention the pregnant bat in the manuscript because it is not included in results. We mentioned the pregnant bats in the methods because we anticipated a reviewer would ask why one group had one less bat than the other groups. We explain this in more detail in line 263-266 "*We determined that one bat in the NP-CGG group was pregnant at the time of the immunization experiments and was immunized subcutaneously to avoid harming the fetus. The pregnant bat was not included in analyses unless stated otherwise. All bats were fed a diet of fruit with a supplement of ground Mazuri Softbill Protein powder*".

Line 270: Was the amount of fruit provided / consumed standardised between groups? Could it be that in the absence of protein supplements (which may have a satiating effect), bats consumed more, and therefore simply had more calories available? That would imply that it is not specifically the lack of protein leading to better antibody responses. Were there any physiological changes (e.g., weight gains)?

R7.b: The amount of fruit was standardized, and we have clarified this (see lines 268-269) and have included a photo of a representative meal (supp fig 4.). We did not include weight data because we have another publication in our lab that is focused on weight as part of broader diet related changes. We can provide those data, if needed.

Line 580 - 582 (supplement): Significantly more detail on the mixture model is needed for me to evaluate this approach. Similarly, the bootstrapping procedure is not described at all in the methods or supplement.

No code was available for review.

R8.b: Code and CSVs were made available for reviewers here: https://zenodo.org/records/10064315 as stated in the manuscript and in the submission portal on PNAS.

Please make the raw data available in machine-readable form (e.g., spreadsheets or csv files). This includes the sequence data (and diversity measures, if reproducible code to calculate these are not provided). Without the sequence data, the data availability statement needs correction as it currently claims all data are provided in the manuscript.

R9.b: All sequences were provided to reviewers (See R8.b).

Minor comments:
Line 68: Sentence truncated.
Line 70: At least one word is missing after "we performed four..."
Line 100: Abbreviation "sub-q" has not been defined.
Line 100 - 101: Check grammar.

###

First round of reviewer comments:

We greatly appreciate the positive feedback and thoughtful critiques provided by the reviewers. Their suggestions have helped strengthen and clarify the manuscript considerably. We have carefully considered each comment and made changes accordingly.

Although we agreed with the reviewers' critiques in most cases, there were a few instances where their recommendations were not feasible or within scope. In each case, we present the rationale and justification of our responses. In these cases, the reviewers' comments helped us identify areas that needed more explanation.

Below, we provide our responses to each of the Reviewers' Comments. We provide the original reviewers' comments, followed by our replies indicated by an "**R#:**".

<u>Reviewer 1.</u>

This study by Crowley and colleagues reports a series of interesting experiments assaying the humoral immune response between Jamaican fruit bats (JFBs) and mice, and the impact of dietary nutrition on the antibody response in bats. Bats and mice were immunised with either KLH (T-dependent antigen), NPCGG (T-dependent antigen) or NP-ficoll (a type II T-independent antigen), and the antibody response assayed by indirect ELISA or competition ELISA. In the second part of the study, JFBs were challenged with Nipah-riVSV or infected with H18N11, with bats receiving either the standard diet or a low protein diet. The antibody response to Nipah-riVSV challenge was assayed by Pseudotyped Virus Neutralization Assay, and the B-cell response to H18 infection studied by BCR sequencing.

Overall, the authors attempt to tackle an important and open question in bat biology – on the nature of the antibody response in bats, and whether bats produce a comparatively weaker antibody response than other mammals upon challenge with a similar antigen. The results of such a line of questioning have important implications to bats as a reservoir for zoonotic diseases and in understanding and predicting disease spillover kinetics. However, the utility of the results of this study to the research community could be greatly improved from substantial changes to the current study design. I would strongly suggest to:

> **R1:** We appreciate the reviewer's positive opinion of our work. We have thoroughly considered the reviewer's feedback and addressed their concerns. We provide detailed responses to the feedback below.

Ensure serological assays for antigens used in Expt 1 and 2 are assayed for both JFB and mouse, for the same time points, and displayed in adjacent panels so that the reader can easily draw conclusions on the response between the two species, for both T-dependent and T-independent antigens. This is not possible with the current data display format and incomplete/imcompatible assays.

R2: We agree with the reviewer that displaying both sets of data will allow readers to interpret the differences between the assay methods and species more easily. We have updated Figure 1 to present the results from the indirect and competition ELISA for bats and mice side-by-side.

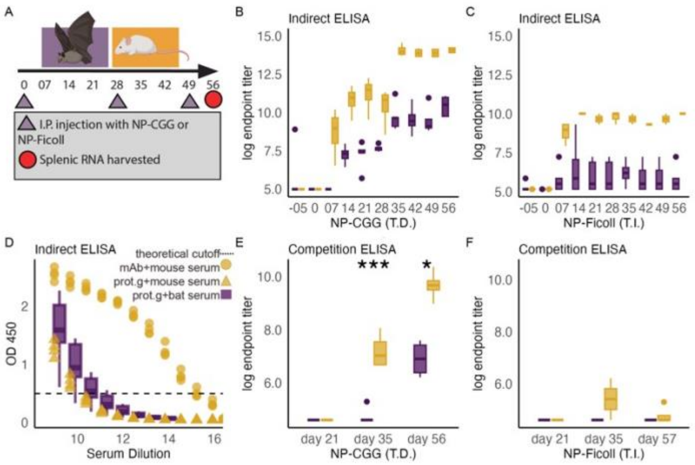

Extend the bat vs mouse immunisation experiment to a viral antigen (Nipah glycoprotein or H18 subunit, for example), which enables pseudotype virus neutralisation assays to be performed between JFB vs mouse serum, independent of species-specific reagents. Having this assay format as a functional readout enables side-stepping the problems the authors have with directly assaying various aspects of antibody response between bat vs mouse in Expt 1 and 2.

R3: We chose these antigens because 1) we wanted to compare T-D and T-I antigens and 2) NPFicoll and NP-CGG are utilized widely in the B-cell field. Since it has been proposed bats undergo limited SHM compared to other, model, mammals we think this was the appropriate immunization strategy to take.

Per the reviewer's suggestion, we agree it is a promising solution to avoid species specific reagents. Had we not been interested in T-D and T-I immunization routes, we might have chosen this option. Now, having developed the competition ELISA, it is not clear to us what new information a pseudotype virus neutralisation assays provides for this paper. Furthermore, it is not clear what new information it would provide that is not in the following papers:

Middleton, D. J. et al. Experimental Nipah Virus Infection in Pteropid Bats (Pteropus poliocephalus). J Comp Pathol **136**, 266–272 (2007).
Williamson, M. M. et al. Transmission studies of Hendra virus (equine morbillivirus) in fruit bats, horses and cats. Aust Vet J **76**, 813–818 (1998).
Williamson, M. M., Hooper, P. T., Selleck, P. W., Westbury, H. A. & Slocombe, R. F. Experimental Hendra virus infection in pregnant guinea-pigs and fruit bats (Pteropus poliocephalus). J Comp Pathol **122**, 201–207 (2000).

Extend the dietary model to calorie shortage, rather than protein deprivation, unless the authors have very strong evidence from literature or bat biology demonstrating specific protein deficiency occurring in the wild in JFBs. This would make this study more relevant to understanding disease spill-over events from bats observed during food shortages (Eby et al, Nature, 2023).

R4: We agree that considering calorie shortages could be informative. However, we were unable to do a calorie restriction:

Our IACUC committee would not allow caloric restrictions. This is in part because bats (as social animals) cannot be housed individually, and if food is limited within a group, dominant bats would eat and non-dominant bats would starve. Moreover, the bats receive all of their water from their food, and rapid dehydration is a risk.

In addition, protein restriction reflects the conditions experienced by adult bats during food shortages. During acute food shortages, nectar and protein-rich pollen from native forests is not available and the bats switch to feeding on weedy introduced species that have abundant fruit that is low in protein (e.g. camphor laurel that grows as a weed across the subtropics of Australia) or orchard fruits such as citrus that also have lower protein content (Eby et al. 2023, Markus & Hall Wildlife Research (2004)). Wood et al. Frontiers in Zoology (2023) presents similar findings in South Africa.

We have added additional language accordingly on lines 157-163: *"Removing Dietary Protein Improved Neutralization of Nipah-riVSV. In Australia Pteropus bats were observed switching from a high protein pollen diet to a low protein fruit diet during winter months, the season in which Hendra virus spillovers occur (refs: Eby et al 2023 & Markus & Hall 2004). Because of this diet switching, we tested the impact of a low protein diet on antibody production in response to Nipah-riVSV (Fig.2A).*

While we agree with the reviewer on the value of further experiments with calorie restrictions, we believe that performing a calorie restriction is currently is beyond the scope of this work and does not compromise the validity and significance of our findings.

Major points

1. In Expt 1 and 2, upon NP-CGG immunisation, blood was reported to be collected weekly from Day 0 (line 233, 242). However, in Figure 1A, the titres at Day 7 are not plotted on the chart. Is this because the JFB titres at Day 7 did not have a significant response compared to control serum? If so, this is useful information that should be displayed. If, however, serum titres were not tested on Day 7 in JFB bats and the earliest time-point assayed was Day 14, it would not be possible to conclude a "delayed response" compared to mice, as stated in Line 90 ("JFB Develop Delayed and Reduced Antibody Responses to T-dependent Antigens"), and the sub-heading should be adjusted accordingly.

    R5: Endpoint titers on day 7 were undetectable in bats, thus it was "NA" in our dataset. We have now included day 7 values in Figure 1.

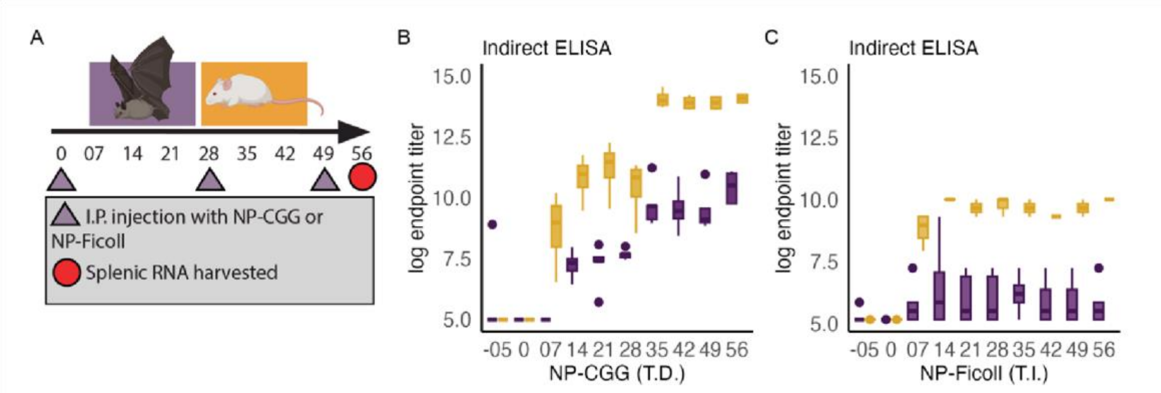

2. If the "endpoint titre was sensitive to the secondary reagent and could not be used for crossspecies comparisons" (line 93), then please remove the phrase "reduced antibody responses" from the sub-heading in Line 90.

> **R6:** We removed the subheading (line 110). Bats did generate a worse antibody response, but we show that inferences cannot be made from results of an indirect ELISA but can be made using a competition ELISA. This information is now contained under the subheading "**Bats Develop Delayed and Reduced Antibody Responses to T-dependent Antigens (line 93)"**

1. Please explain why indirect ELISA results are not shown for (JFB vs) mouse immunisation with KLH and NP-Ficoll as these antigens are described in Expt 1 and 2

> **R7:** In the pilot study we used KLH and NP-Ficoll. The indirect ELISA data are in supplement figure 1C and 1D. When we repeated the study and included mice, we decided to use NP-CGG as our T.D. antigen because the route of immunization is the same (I.P.) and both NP-CGG and NP-Ficoll share the 4-Hydroxy-3-nitrophenylacetyl (NP) group. This enabled us to conduct a competition assay. We have modified the text to make this clearer, please see lines 93-98:
>
> ***Bats Develop Delayed and Reduced Antibody Responses to T-dependent Antigens*** *We next immunized both bats and mice to provide a species comparison (Fig.1A). For this experiment, we utilized an I.P. route for immunization of 4-Hydroxy-3-nitrophenylacetyl conjugated to Chicken Gamma Globulin (NP-CGG) (T.D.) in lieu of a sub-q immunization with KLH. We made this change to have comparable immunization routes between the T-dependent immunogen and Tindependent immunogen. Further, we waited longer to boost the animals for the first and second time to assess if bat antibody responses would improve given more time.*

2. Can the authors elaborate why is there a notable difference between indirect ELISA vs competition ELISA data?

> **R8:** An indirect ELISA can be thought of as a function of two separate processes. 1) serum antibodies binding to the antigen and 2) the detection reagent binding these serum antibodies. When conducting species-comparative work, the second issue becomes important to control for. Thus, we developed the competition ELISA which detects the

competitor antibody and avoids the bias introduced by species-specific secondary reagents.

No response at all is detected via competition ELISA in JFBs upon immunisation with NP-Ficoll up to day 56 (Fig 1E), but Sup Fig 1B shows a measurable titre via indirect ELISA for NP-Ficoll in JFB from day 4-43. Similarly, JFB immunisation with NP-CGG only has robust competition ELISA titres on Day 56 (Fig 1E left panel), but indirect ELISA has detectable titres from Day 14-56 (Fig 1B).

R9: Sub Fig 1B shows titers at around 1:50. The data suggests at that dilution there are not sufficient antibodies to knock off the B1-8 competitor antibody.

Can the authors justify the use of 1/100 minimum dilution for bat serum (Line 502)? Were lower dilution factors tested? It would be surprising if such a high minimum dilution does not affect the sensitivity of the competition ELISA.

R10: The text says "For the competition assay, we incubated bat or mouse serum at dilutions of 1 to 100 to 1 to 1.5 x 10^5". We have changed the text on lines 527-536 to clarify that a range of dilutions were tested:

We designed a competition ELISA to compare the antibody responses between JFB and mice. ... For the competition assay, we diluted immunized animal serum from a 1:100 dilution to a 1:1.5 x 10^5 dilution. This dilution series of serum was combined with a single dilution of rabbit anti-NP monoclonal antibody B1-8 (Abcam ab289966). The dilution of B1-8 was 2 µg/µL for NP-CGG or 4 µg/µL for NP-Ficoll tests. ELISA plates were coated with NP-CGG or NP-Ficoll in sodium bicarbonate at a concentration of 0.5 µg/mL. The serum and B1-8 mAb mixture was added to the plates and incubated for two hours at room temperature. For the NP-CGG assays, detection was done using an antirabbit monoclonal antibody. For the NP-Ficoll assays, detection was done by biotinylating (Abcam ab201795) the B1-8 mAb and using avidin-HRP at a 1:5000 dilution as our detection reagent.

3. For experiment 4 (H18N11 virus infection), the bats were placed on standard diet or low protein diet for 3 weeks before virus inoculation. For experiment 3, which is testing a similar effect of diet on antibody response to Nipah-riVSV challenge, were the bats pre-adapted to the two diets similarly? If so, this information needs to be included in the Methods section for Experiment 3, line247 onwards.

R11: We have made changes for clarifications as follows: Figures (Fig 2A and 3A) and in text:

Lines 262: ***Experiment 3: Food Restriction with Nipah-riVSV*** *To assess if diet affects the antibody responses of JFBs, we assigned a total of eight bats to one of two dietary regimes. The first group received a fruit-only diet (n = 4, two males and two females). The second group (n = 4, two males & two females) received the standard diet (e.g., honeydew, cantaloupe, watermelon, bananas, strawberries, orange with a supplement of ground Mazuri Softbill Protein powder). Bats began their diets two days prior to challenge.*

Fig 2A:

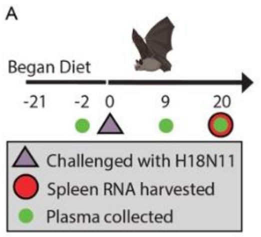

Lines 278: ***Experiment 4: Food Restriction with H18N11 virus Infection*** *We designed a second food manipulation experiment to assess the effects of diet on the immune response of bats and viral shedding. We used the H18N11 influenza A-like virus for which Artibeus bats are a reservoir* [31,58]*. We assigned eight bats each to the two diet regimes described previously. The first group received the fruit-only/low protein diet (n = 8 males). The second group received the standard diet (fruit supplemented with Mazuri Softbill protein powder) (n = 8 males). Bats were placed on their diets for three weeks. After three weeks, six bats in each group were inoculated intranasally with* 5×10^5 $TCID_{50}$ *of H18N11 virus.*

Fig 3A:

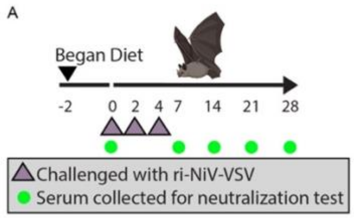

4. Why was "low protein" diet selected as the dietary change? Is there evidence from prior studies that fruit bats encounter periodic changes in protein availability in their diets?

    R12: Please refer to **R4.**

5. It is important to present some data on the food intake per bat during standard versus low protein diet, to understand whether the observed effect on antibody titre is a result of protein deficiency or calorie intake. For example, an increase in overall fruit consumption in the low protein diet in an attempt to compensate for the lower protein content by the animals could conceivably increase total calorie intake in this treatment group.

    R13:

    Food was provided *ad libitum*. We weighed the remaining food each day in the cage. We did observe that bats on the fruit consumed more food, but that effect is explained once we controlled for the body weight differences by cage. We can provide this information, analysis, and data to reviewers but plan to include these data in another manuscript.

Minor points

Abstract Lines 26-28: “Moreover, bats generate low neutralizing antibody titers in response to experimental infection, although more robust antibody responses have been observed in wild caught bats during times of food stress.” Assuming this refers to prior studies, this sentence should be moved to the introduction or discussion section, and backed with relevant citations.

> **R14:** We have followed the suggestion by moving the sentence and including the relevant references sustaining it (lines 38-54).

Abbreviations for KLH, IFA, NP-CGG, NP-Ficoll etc need to be described on the first occasion of use
R15: Done. See lines 78-95.

Line 501 has a missing word (Protein G, a commonly used secondary reagent for detecting bat antibodies exhibits? species specific Ig affinities)

> **R16:** We corrected the sentence. Line 528: “*Protein G, a commonly used secondary reagent for detecting bat antibodies, possesses species-specific Ig affinities, biasing results even within a genus*”.

<u>Reviewer 2.</u>

Comments on Significance Statement:

As noted below in my general comments, the authors need to be careful to reference zoonotic viruses only, rather than pathogens in general, since this is the focus of their study. I might recommend stating that bat antibodies have "lower affinity" rather than "worse binding capacity" than mice. Stating that "if you remove protein from their diet" sounds a bit childish - maybe "if protein is removed from their diet" would be better? And rather than saying that "antibodies improve", it should be stated that antibody binding (or affinity) improves. I would also add to the last sentence that these are "spillover events for high profile zoonoses".

> R17: We agree and can incorporate these suggestions.

Comments:

Bats are known reservoirs for important viral zoonoses, and the literature has shown a pattern of heightened seroprevalence for several bat-borne viruses during periods of low nutrient availability for wild bat populations. Moreover, several authors on this manuscript have also demonstrated that seasonal food shortages for Australian fruit bats have led to increased shedding of Hendra virus from bats into the surrounding environment, and in some cases, driven spillover of Hendra virus to humans. Because of the hypothesized interaction between bat nutrition and immunity, this team seeks to test the effects of diet manipulation on antiviral antibody responses in captive Jamaican Artibeus jamaicensis fruit bats (hereafter, JFBs).

Several previous studies have shown that bat antibody responses are both delayed and at lower affinity than those typically mounted by other mammals in response to viral infection. It has been hypothesized

that these low affinity antibody responses could be the result of a perceived pattern of higher diversity in B cell receptors (BCRs) for bats, which might drive more generalist, lower affinity antibody responses that could also minimize bats' reliance on somatic hypermutation (SHM).

The authors address this question with four experiments, which I summarize below, with comments on the comprehensibility of the approaches and validity of the results presented. In general, I am enthusiastic about the conceptual underpinning of this manuscript-it represents an enormous amount of work and several important findings, chiefly presenting evidence that high BCR diversity may tradeoff with low antibody affinity in JFBs and that low protein diets may shift this balance back towards lower BCR diversity and higher antibody affinity. However, the paper is very difficult to read and presented in a somewhat convoluted order, jumping around between experiments and panels of figures in different orders, and I spent a very long time trying to interpret it. There are also several figure panels that are not discussed anywhere, and some results presented without methods to demonstrate that where they came from, as well as a few experiments mentioned with no follow up or data presented. This challenging read greatly diminished my enthusiasm for the overall manuscript, though I am still excited about the study.

> **R18:** We greatly appreciate the reviewer's thoroughness of feedback provided. After detailed consideration of each of the reviewer's comments, the revisions have improved the manuscript.

Experiment 1:

They first undertake a pilot experiment to test the timing and efficacy of JFB boosting of humoral immune responses. Here, they challenge 15 male bats in two groups with either (group 1) KLH + IFA (a T-dependent antigen that should elicit an immune response characterized by SMH and affinity maturation; administered sub-Q) or NP-Ficoll + SS (a T-independent antigen that should not elicit SMH; administered IP), then measure end-point antibody titers to the various antigens using an indirect ELISA across the time course of the experiment (which includes boosting on day 21 and day 42). At day 43, they euthanize the bats and harvest spleen for qPCR and 5'RACE of BCR. FigS1B shows (qualitatively) higher endpoint titers for the KLH-IFA (T-dependent) treatment after boosting on day 21, those these results are not discussed in the manuscript anywhere, and no statistical evaluation is presented. The authors do briefly discuss that (and show in Fig S1C) that they find no difference in BCR diversity between the two treatments for IgHM and marginally decreased diversity in BCR IgHG sequences for the KLH+IFA (Tdependent) treatment. They present no statistical evaluation of the diversity differences across the two treatments, only the visual results from Fig S1C. Since the T-dependent antigen is supposed to be associated with SHM, high affinity antibodies, and lower BCR diversity, this results seems a bit contradictory to the rest of the manuscript.

> R19: We were concerned including a statistical test would give readers the impression that mice and bats' indirect ELISA results could be compared. Please also see R22.

I conclude that this experiment needs to have its purpose, hypothesis, and results more clearly defined. I do not believe it is acceptable to present a figure (Fig S1C) without interpretation in a scientific publication.

> **R20:** We have added text for figure S1C in lines 86-91. We have added more clarification surrounding experiment 1 in lines 78-80.

Experiment 2:

In experiment 2, the authors compare antibody responses of 12 female bats with 8 female BALB/c mice, with subgroups of each species immunized with either the same (a) NP-Ficoll-SS (a T-independent antigen) as in experiment 1 or (b) NP-CGG (a T-dependent antigen different from that used previously). The authors do not explain why they orchestrated the shift from immunization with KLH-IFA to NPCGG (maybe to resolve inconsistency in route of inoculation from the first experiment?). All animals were immunized (I.P.) on day 0 and boosted on days 21 and 48, and blood was collected for serological analysis periodically throughout the experiment. One bat in the NP-CGG group was found to be pregnant at the point of immunization and was left out of most statistical analyses. It is stated in the methods that she was immunized I.P. to avoid fetal harm, but since this was the method presented for the whole experiment, it suggests there is a typo somewhere.

R21: We have added more language to explain why we used NP-CGG in lieu of KLH for the mouse and bat side-by-side experiment. **Lines 93-99:** ***Bats Develop Delayed and Reduced Antibody Responses to T-dependent Antigens*** *We next immunized both bats and mice to provide a species comparison (Fig.1A). For this experiment, we utilized an I.P. route for immunization of 4-Hydroxy-3-nitrophenylacetyl conjugated to Chicken Gamma Globulin (NP-CGG) (T.D.) in lieu of a sub-q immunization with KLH. We made this change to have comparable immunization routes between the T-dependent immunogen and T-independent immunogen. Further, we waited longer to boost the animals for the first and second time to assess if bat antibody responses would improve given more time.*

The pregnant animal was immunized sub-cutaneous and that was a mistake. We appreciate your careful reading and we have fixed that.

As in experiment 1, animals were euthanized at the end of experiment 2 (here, day 56), and spleen was harvested for qPCR and 5' RACE of BCRs. The authors qualitatively demonstrate that endpoint AB titers were higher in mouse vs bat responses to NP-CGG (the T-dependent antigen) in Fig1B; no data are presented for the endpoint titers to NP-Ficoll (the T-independent antigen), which adds to my confusion as to what these T-independent experiments are supposed to show (same from experiment 1). The authors conclude that the endpoint titer of their ELISA is sensitive to a secondary reagent and therefore not directly comparable across species - again, data are only shown for this bias in the case of NP-CGG treatments. No methods are presented for this determination (only Figure 1C), so it is difficult to understand what exactly was assessed. I think that the authors are trying to show that the end point titers are significantly different between JFB serum when the secondary reagent is protein G vs. mouse serum when the secondary reagent is anti-mouse IgG. It looks like there is no significant difference between JFB + protein G and mouse + protein G, so it makes me wonder why they didn't just use protein G consistently... Perhaps this approach was impossible for some reason, but it is not presented in the methods. Additionally, the notation for the secondary reagents in Fig 1C is different from that in the methods (L485) - these should be consistent to make more comprehensible.

R22: You are correct that we only show that the endpoint titers are qualitatively higher. This was intentional as we do not want readers to mistakenly conclude one can compare the endpoint titers.

Per the comment "It looks like there is no significant difference between JFB + protein G and mouse + protein G, so it makes me wonder why they didn't just use protein G consistently..."

This is the bias we are trying to avoid. If we use protein G, we conclude mice and bats have the same OD450. If we use a mouse specific mAb for the mice, we then conclude mouse and bats are quite different. One cannot compare them. We changed figure 1D and hope this makes it clearer:

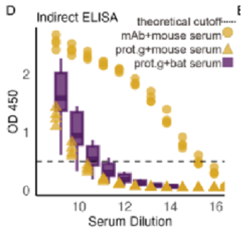

We think this is an important point for readers, because we often see reports of 'low titers' in bats, but if this conclusion comes from an indirect ELISA then this is a mistake.

In response to this detected bias in the endpoint titer results, the authors then develop a competition ELISA to enable direct comparisons between bat and mouse. They have a diagram of this assay in Fig1D, but caption or methods could use some more detail, as it was difficult to understand how it worked. My takeaway after rereading several times was that they incubated bat or mouse serum at different concentrations with a competitor antibody (B1-8 rabbit) with either NP-CGG of NP-Ficoll, then measured the ability of the tested serum to displace the competitor. Higher concentrations of serum should more easily displace the competitor, and presumably, higher affinity antibodies should be able to displace the competitor even at lower concentrations of serum. The authors present the result that, post-boosting for NP-CGG (the T-dependent antigen), mouse serum displaced the competitor antibody on day 35 and 56, while the bat serum did not. It is unclear from the text at what dilution this result was recovered. They also separately state that mouse serum could displace the competitor AB on day 28 (L102-103) at dilution 1:50 but these results are not shown in the graph (no bar for day 28). I am not sure how to interpret the yaxis of Fig 1E and connect it to the dilution needed to displace the antibody. The authors further state that JFB serum could not displace the competitor antibody under NP-Ficoll (T-independent) challenge at any timepoint. From the graph, it looks as though mouse was able to do so on day 35 and the signal was lost (for reasons that are not explained) on day 56. This also needs explanation in the text, as it is only perceptible from the figure.

R23: We agree that the competition ELISA could benefit from a larger visual. We are expanding the visual of the competition ELISA in supplemental figure 3.

This is the current iteration of supplemental figure 3:

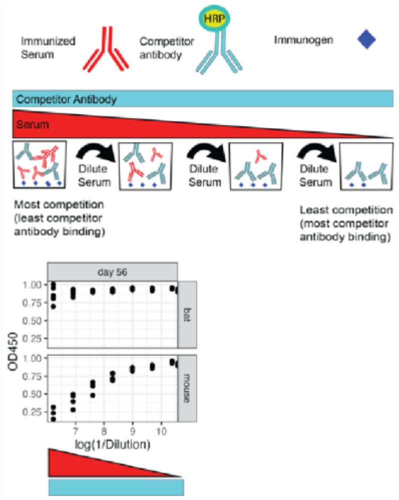

The Y axis of Fig 1E is a log endpoint titer of a competition ELISA. We have added new language in lines 299-301 to clarify this: *We designed a competition ELISA to compare the antibody responses between JFBs and mice (Fig.1C and Supp. Fig. 3) as described in SI methods. This competition ELISA was designed to report and endpoint titer that was not biased by secondary reagents for mouse and bat antibodies (Fig.1B)*[61]. And in lines 530-539:
We designed a competition ELISA to compare the antibody responses between JFB and mice. This competition ELISA was designed to minimize bias introduced by secondary reagents for mouse and bat antibodies (Fig.1D). Protein G, a commonly used secondary reagent for detecting bat antibodies, possesses species-specific Ig affinities, biasing results even within a genus [61]***. For the competition assay, we diluted immunized animal serum from a 1:100 dilution to a 1:1.5 x*** 10^5 ***dilution.*** *This dilution series of serum was combined with a single dilution of rabbit anti-NP monoclonal antibody B1-8 (Abcam ab289966). The dilution of B1-8 was 2 µg/µL for NP-CGG or 4 µg/µL for NP-Ficoll tests. ELISA plates were coated with NP-CGG or NP-Ficoll in sodium bicarbonate at a concentration of 0.5 µg/mL. The serum and B1-8 mAb mixture was added to the plates and incubated for two hours at room temperature. For the NP-CGG assays, detection was done using an anti-rabbit monoclonal antibody. For the NP-Ficoll assays, detection was done by biotinylating (Abcam ab201795) the B1-8 mAb and using avidin-HRP at a 1:5000 dilution as our detection reagent****. The endpoint titer was the estimated serum concentration where no displacement of the B1-8 competitor was observed.***

The authors validate this competitor ELISA with an IC-50 assay which measures the 'molarity of the chaotropic agent required to reduce the OD-450 by 50% relative to the control'. In the supplement (SFig 2A), the authors show some linear regression of OD-450 against AT-molarity, using the 2 detection agents, but they do not explain the expectations or results. As someone unfamiliar with this assay, it is difficult to interpret how it shows that the experiment is no longer biased. After studying it carefully, I concluded that they were trying to show that there is no difference in the slope of this line (molarity vs. OD-450) vs. the two detection agents used (goat anti-mouse IgG or protein G) at different timepoints. This could be incorrect, and either way, no statistics or regression coefficients are presented to show that the lines do not differ. In the main text, an R2 for the linear regression is presented, but it is not clear whether it refers to the regression shown for protein G or anti-mouse IgG (or both).

R24: We've removed these plots. For context, the IC50 assay required months of optimizing, but in retrospect do not provide additional information. The assay has limitations, which is why the figure is split into two graphs. Pre and post boosting, we concluded from optimizing the assay

that the IC50 results are not comparable pre and post boosting, so we split the results into two graphs.

NP-CGG immunized bats were used to validate assays instead of NP-Ficoll immunized bats. This was because the NP-Ficoll, being T-independent, generates weak titers. Using this NP-Ficoll serum to optimize the assays would have utilized most of the serum and left little serum for the assay itself.

The authors also present direct results from the IC-50 assay in SFig2B, which shows that mouse serum had a higher IC-50 (which I am interpreting as a signal of displacement ability against the competitor AB) in NP-CGG challenge after initial immunization on days 14, 21, 28 and at all timepoints after boosting. Bat serum is unable to displace the competitor AB at all timepoints for the NP-CGG challenge. No results are shown for NP-Ficoll challenge, with no explanation given. Additionally, the authors report IC-50 values for KLH-IFA immunized bats in experiment 1 in Fig S2c, but no interpretation of these results is provided. This also needs addressing.

R25: See R24

Finally, the authors compare BCR sequence diversity from harvested splenic mRNA in bat vs. mouse from experiment 2. They show that bats had higher BCR diversity (by several metrics) than mice for both NP-CGG and NP-Ficoll challenge and that differences were most clearly observed in IgHM but also present in IgHG. They do not present anywhere how they are able to distinguish between these sequence classes. Is this a simple thing? My naïve understanding was that this distinction is challenging for bats.

R26: While it is a simple bioinformatics step, we agree this would not be clear to most readers. We added new text in lines 305-311: *We processed, filtered, and analyzed the sequencing BCR reads using pRESTO and previously described germline free identification methods, as described in SI methods [63,64]. **Bat IgG and IgM sequences were separated using pRESTO by primers listed in Supp. Fig. 2A.** Briefly, the TF-IDF (term frequency, inverse document frequency) quantified the distance between BCR sequences. We tested the number of nucleotides from the 3' end of the BCR sequence to use in analyzing sequences (Fig.2B). We used hierarchical Bayesian mixture models in Rstan 2.21.8 [65] to bin sequences into clusters ("clones") using Hill Diversity Curves [66–68] (Fig.2C). Statistical significance tests of the Hill Diversity Curves were done at the species level as described previously [66].*

Supp Fig 2A:

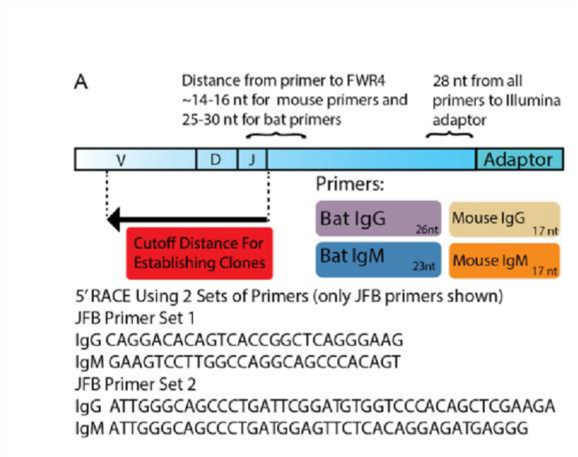

In addition, Figure 2E shows BCR diversity results in spleen as well as mesenteric lymph nodes (MLN) but it is not stated in the methods anywhere that MLN were harvested or sequenced in experiment 2 (though apparently they were). It is mentioned that large MLNs were observed in bats during necropsies though only qualitative information is presented. If these were harvested for sequencing, it should be included in the methods.

> **R27:** It is now mentioned in the methods. We modified the language to communicate the findings as a qualitative observation. The size is qualitative and we now state this.
> The manuscript now states on line 138: "*During necropsies, we harvested lymph nodes from mice and bats. We collected lymph nodes that were visually obvious and recorded the number of each lymph node type we harvested (Supp. Table 2).*

Experiments 3/4:

Finally, the authors undertake two diet experiment in which they assign JFBs to two different treatments: fruit only or fruit + supplemented protein and challenged them with (experiment 3) a replication incompetent NiV-rVSV or (experiment 4) a real virus: replication-competent H18N11 influenza A-like virus, for which JFBs are the known reservoir hosts. In both cases, bats were fed the two diets, then challenged, then blood was collected at various time points following challenge for serology. For experiment 4, 3 individuals per treatment plus one negative control from each diet group were sacrificed, and RNA was harvested from spleens and MLNs for BCR sequencing. I believe this last step was at the end (after day 20), but this is also not stated.

> R28: We have added more information on when animals began their diets, and that spleens and lymph nodes were harvested only after euthanasia. Please see R82 and R83 for more information.

The NiV-rVSV challenge resulted in differences by diet: bats fed only fruit developed a neutralizing titer on day 7, which increased consistently through to day 28, while those fed a protein supplemented diet never developed a neutralizing antibody titer. The authors observed that bats on day 28 in the proteinsupplement diet actually had a significantly lower neutralizing titer as compared to those on day 0.

Similar results were obtained for the H18N11 challenge. The authors tested antibody responses to the H18 glycoprotein across the timepoints of experiment 4 using two probes, a F4 mAb, (which they indicate binds an Ig light chain), as well as protein G. They measured significantly higher plasma OD450 in fruit

vs. protein diet bats when probing with F4 mAB but no differences when probing with protein G. They do not discuss the implications of these differences anywhere or why they chose to use these two different probes.

R29: We appreciate you bringing this to our attention. We have added new text in the discussion and results. See 192-202: *Our findings also suggest the diet of bats has a direct impact on their antibody response and can be manipulated to improve their antibody response. Antibody titers correlated with BCR sequence diversity from the MLN tissue in the food restriction experiment. Our findings shed light on the associations between body condition, food availability, and antibody metrics observed in wild bats. Previous work in bats demonstrated a connection between changing environmental conditions with pathogen shedding and zoonotic spillover [40,41]. Our findings suggest that the food bats consume directly impacts their antibody response and offer a mechanistic connection between the quality of antibody responses in bats and ecological conditions**. Interestingly, the diet induced changes were observed only with an anti-light chain antibody, not protein G, suggesting an antibody class other than IgG was responsible for this change. It remains unclear what antibody was impacted by the diet which impacted NipahriVSV neutralization.***

Sequencing results from experiment 4 showed few differences across diet treatments in experiment 4, but the authors did observe higher IgHM sequence diversity in MLN mRNA in high protein diet bats (corresponding to their less robust antibody responses). No results of the sequencing data for the control bats are presented. These should be included in the paper.

R30: We added new text to explain why negative controls were not included in the Hill Diversity Curves (Line 501). The negative controls had very few sequencing reads, likely because they are not mounting a B cell response. When there are very low read counts the Hill Diversity Curves are not reliable. We included new wording of this and the appropriate citation.

Overall takeaway:

This paper describes some stellar work but the manuscript could do with pretty thorough redesign. My recommendation would be to start by writing a supplementary document in chronological order that explains each experiment, its methods, and its findings. After this, in the main text, I would suggest that the authors give a brief summary of all 4 experiments at the end of the introduction, including their hypotheses for each and main findings; a conceptual summary figure could help with this too. I would suggest that they then present the results in the same order as the experiments were conducted, making sure to discuss all results.

R31: We restructured the paper per your suggestions and believe it is now a much clearer manuscript. Specifically, we have split each experiment into it a stand-alone figure and present the work chronologically. We also include a conceptual summary of each experiment in part “A” of each figure. See Fig 1 for example:

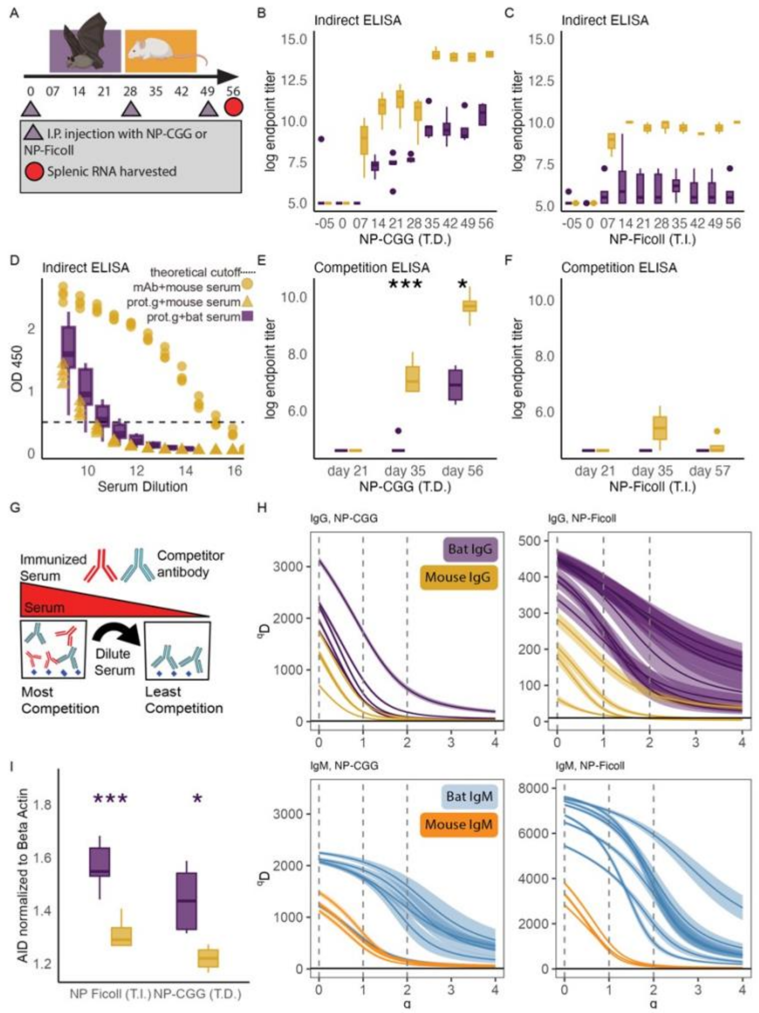

Figure 1: Experiment 2; Mouse and Bat Side by Side Experiment Show Bats Develop Low Affinity Antibodies and have more BCR Diversity in their Spleens; A. Timeline of immunization, boosting, bleeding, and tissue collection. **B**. Indirect ELISA endpoint titers from bats and mice immunized and boosted with NP-CGG (Tdependent). **C**. Indirect ELISA endpoint titers from bats and mice immunized and boosted with NP-Ficoll (Tindependent). **D**. Indirect ELISA dilution curve (with endpoint titer indicated as a dashed line) from bats and mice immunized and boosted with NP-CGG. Serum is from day 56. Bat serum IgG was measured using protein-G as the secondary reagent. Mouse serum IgG was measured with either protein-G or a monoclonal antibody specific to mouse IgG as the secondary reagent. The dashed line at 0.5 indicates a theoretical OD450 cutoff to establish an endpoint titer. Serum dilution is shown on a natural scale. **E**. Competition ELISA endpoint titers from bats and mice immunized and boosted with NP-CGG (T-dependent). **F**. Competition ELISA endpoint titers from bats and mice immunized and boosted with NP-Ficoll (T-independent). **G**. Competition ELISA visual schematic. To avoid the bias of the secondary ELISA reagents, a competitor antibody was kept at a single concentration while immunized serum was diluted. As the animal serum is diluted out, more of the competitor antibody can bind to the immunogen. A more detailed schematic is shown in Supplemental Figure 3. **H**: Splenic B cell receptor IgG (top

panels) and IgM (bottom panels) mRNA diversity from bats and mice against NP-CGG (left panels) and NP-Ficoll (right panels). Higher ^{q}D values indicate greater diversity within the sample. When q equals 0, the ^{q}D value reflects the total count of unique B cell receptor sequences within the dataset. When q equals 1, ^{q}D reflects the Shannon Diversity measurement. When q equals 2, ^{q}D reflects the Simpson Diversity measurement. Each line represents an individual animal, and a ribbon depicts the 95% CI, generated by bootstrapping. Overlapping ribbons indicate no significant differences between individuals. **I.** Splenic AID mRNA expression levels normalized to beta actin for NP-CGG (right) and NP-Ficoll (left). *Boxplots show the 25%, 50%, 75% percentiles, lines indicate the smallest and largest values within 1.5 times the interquartile range, dots indicate values beyond that. When an endpoint titer could not be estimated, the value was set to that of the pre-immunization serum. P values derived from F statistic. Significance codes: '***' **P** < 0.001 '**'<**P** 0.01 '*'**P** < 0.05.*

I summarise a few other general points here:

1. It would be helpful if the figures could consistently reiterate to the reader what is the T-dependent vs independent antigen, rather than (or in addition to) using the antigen names. Color coding this would also help.

R32: We have now included T-independent and T-dependent on figures and in text. Figure 1, for example:

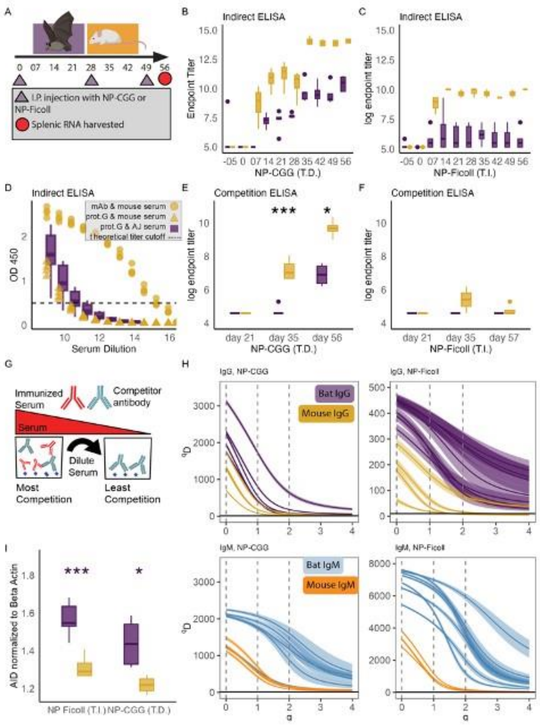

2. The figure captions need additional detail, as many of the figures are hard to understand and the axes not explained. In particular, nowhere in the paper do the authors explain what the hill diversity plots show - what is qDiversity on the y-axis vs. q on the x-axis? I can generally understand that the colored lines that are higher correspond to higher diversity estimates, but the captions or methods (or both) could make these much more interpretable.

R33: Done. We have expanded all figure captions, paying particular attention to explaining axes, symbols, and measurements presented. For the Hill diversity plots we now explain in the caption that the y-axis shows the qDiversity index, which provides a measure of repertoire diversity based on the q value input. We also explain key q values in the caption (q=0 as richness, q=1 as Shannon diversity, q=2 as Simpson diversity). Additional explanation of how to interpret the Hill diversity plots is also now provided in the main text. For example, the text in figure 1 now states: *"**H**: Splenic B cell receptor IgG (top panels) and IgM (bottom panels) mRNA diversity from bats and mice against NP-CGG (left panels) and NP-Ficoll (right panels). Higher ^{q}D values indicate greater diversity within the sample. When q equals 0, the ^{q}D value reflects the total count of unique B cell receptor sequences within the dataset. When q equals 1, ^{q}D reflects the Shannon Diversity measurement. When q equals 2, ^{q}D reflects the Simpson Diversity measurement. Each line represents an individual animal, and a ribbon depicts the 95% CI, generated by bootstrapping. Overlapping ribbons indicate no significant differences between individuals."*

In the main text we also include more clarification. For example (lines 91-107):

We further assessed the B cell receptor (BCR) diversity in the spleen using Hill's Diversity Curves. Hill's Diversity uses input values (q) to generate a ^{q}D index of diversity. Q ranges from 0 to infinity with interpretable outputs at specific values. When q=0, ^{q}D is richness (the total number of unique B cell receptor sequences), when q=1 then ^{q}D is the Shannon Diversity measure, and when q=2

then ${}^{q}D$ is a transform of the Simpson Diversity measurement (the probability that two randomly selected BCR sequences belong to the same B cell receptor sequence). We observed … (Supp. Fig. 1E).

3. A conceptual figure summarizing experiments at the beginning would free up some space in the rest of the figures (as panel 1A and SFig1A could be dropped). I also think that the bioinformatics methods of sequence diversity determination by clone binning could be moved to the supplement (e.g. Fig 2A,B,C and 3D) as could the end-point titer validation (Fig 1C). In several instances, the figures are also a bit blurry (not sure if this is the authors' fault or that of the journal), and they look very much stuck together at random. Given the high profile nature of the journal, these need to be more cleanly ordered and crisply presented.

R34: We have followed the suggestion and by including a summary of the respective experiment on each figure.

We followed the suggestion and moved this content to the supplement. For example, the following figures are now in supplement figure 2:

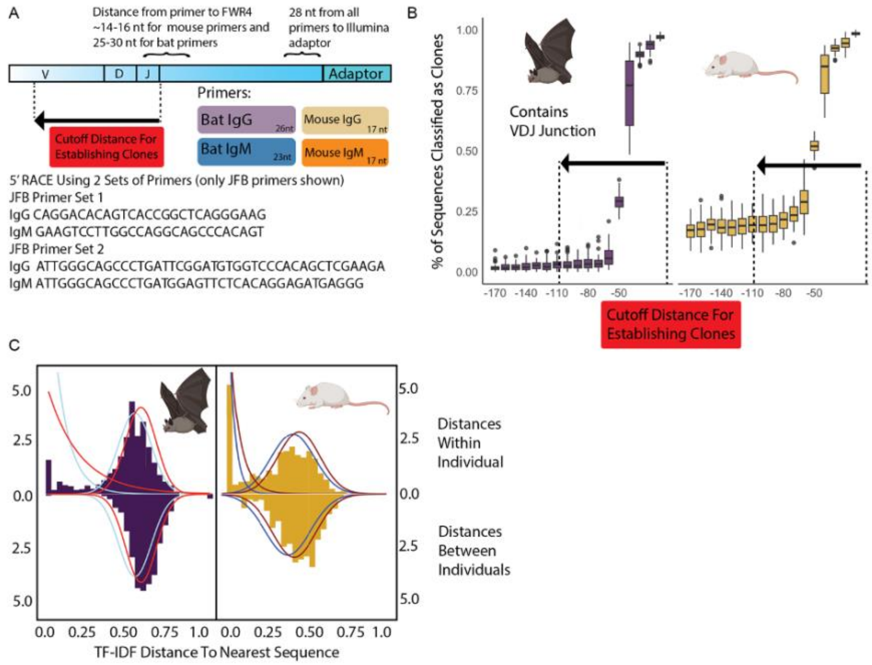

I also think that the bioinformatics methods of sequence diversity determination by clone binning could be moved to the supplement (e.g. Fig 2A,B,C and 3D) as could the end-point titer validation (Fig 1C).

R34: Done. Detailed descriptions of the sequence analysis (clone binning, etc) and endpoint titer validations have been moved to the supplemental information to improve flow of the main text.

In several instances, the figures are also a bit blurry (not sure if this is the authors' fault or that of the journal), and they look very much stuck together at random. Given the high profile nature of the journal, these need to be more cleanly ordered and crisply presented.

> **R35:** Done. We have thoroughly reviewed all main and supplemental figures to improve clarity, sharpness and spacing.

4. They authors show in the figures that mRNA was harvested from spleen for qPCR and 5' RACE for BCR sequencing. No qPCR results are shown or presented anywhere. This should be removed from the figures if not included in the paper.

> **R36:** Done. We added additional qPCR data on AID expression levels. See figure 1I and supplement figure 1B.

5. There is no github repo or zenodo upload that I can see that shares the raw data and code used for analysis. For a journal of this caliber, this is essential for this work to be reproducible. All we have to evaluate as it currently stands are the plots.

> **R37:** We fully agree. The code for the review process will be made available. There are parts of the analysis, particularly related to the processing of raw sequencing data, that require more than 100 hours of computational time on a machine with 24 cores and 128 GB RAM. For replication of these steps for the review process, we can provide a random subset of the data. Replicating the steps of processing of this subset of the data might take approximately 2 hours.

6. The authors do not discuss the impact of reproduction on bat antibody responses. Since they find a pregnant bat in their study, it makes me wonder if there could be female reproductive hormone effects that could impact the last 3 experiments in any way. At a minimum, it would be helpful to acknowledge in the introduction or discussion that this has been described in the literature in several studies on wild bats (including some of those they cite as evidence of impacts of nutrition).

> **R38:** This is an important point, and it is now mentioned in the discussion in lines 214-217. We aimed to conduct all the experiments with only male bats, however this was not possible due to the number of individuals available at the time of the experiments.

7. In the abstract, the authors state that "T cell help to B cells is dampened in bats resulting in low affinity antibodies, but this phenotype can be manipulated with dietary changes". The authors could do a better job of more clearly presenting this hypothesis, as this sentence in the abstract is the only place where the link between T cells and B cells and affinity maturation is made. While this may be obvious to immunologists, the readership of this article is likely to be much broader, and it would be helpful for them to explain in the Introduction (~L68-72) that helper T cells activate B cells in germinal centers which undergo SHM, then T cell selection to result in B cell production of antibodies with higher affinity to a specific antigen (affinity maturation).

> **R39:** Starting on line 65-71 we added text that is more explicit regarding our thinking: *"To directly test whether bats generate lower affinity antibodies than other mammals and to determine if diet may mediate this process, we performed a series of experiments on Jamaican fruit bats (Artibeus jamaicensis; JFB) and BALB/c mice. We immunized both species with commonly used immunogens to measure their antibody response to a non-infectious challenge. We used T-dependent (which elicits a classic response, with SHM and affinity maturation) or*

Tindependent immunogens (which elicit an antibody response, but do not require T cell help and do not elicit SHM or affinity maturation). We hypothesized that bats would generate lower affinity antibodies than mice in response to T-dependent antigens but would generate comparable affinity antibodies to mice with a T cell-independent antigen."

Finally, the paper is riddled with typos, grammatical errors, and, in a few cases, some incorrect citations. I address some in line-by-line comments below.

R40: We have reviewed the manuscript and corrected the errors and considered the references suggested. We have integrated the references when appropriate.

Abstract

L27 - Specify that this is viral infection

R41: We have now specified this in line 20.

L30 - The abstract does not mention experiment 1 or 3. Since these are main components of the paper, they should be mentioned here.You also did NiV-rVSV, need to mention this too. Also should mention experiment 1

R42: Experiment 3 and NiV-rVSV are now mentioned on line 24.

L33 - See general point #7 above. This is the only mention of this interaction in the paper.

R43: Please see R39.

Main text

L42: This virulence claim needs citation support: Guth et al. 2022, PNAS

R44: Done. We have included the citation suggested on line 33.

L46: Persistent infection for bat CoVs is also hypothesized in Jeong et al 2017 Epidemiol. Infect.

R45: Done. We have added this citation on line 35

L48: "and thus facilitating" - grammatical error

R46: Fixed

L50: Only the henipavirus studies are cited here. What about experiments with lyssaviruses (Freuling et al 2009, Sulkin & Allen 1974) and filoviruses (Schuh et al 2017 Sci Reports, Nat Comm; Paweska et al 2012 PLoS One, 2015 JID, etc)? The lit review here needs to be thorough

R47: We appreciate the literature suggested. It certainly is all relevant to bats and their antibody responses. In the original paragraph we referred only to comparative studies. We added additional wording to make this more explicit, but also included the references you mentioned.

Originally, we did not include Frueling et al because it does not compare bats to a non-bat species.

Schuh et al was the third paper we cited. We felt it was an appropriate citation for the potential persistent infection, however we were not sure it is an ideal citation for bats not generating a robust antibody response. First, the paper lacks a comparative species. Second, the authors' gamma irradiated the serum. We have run experiments that show *Pteropus* serum IgG is more sensitive to gamma irradiation than human serum IgG. We do not know why their antibodies appear more sensitive to gamma irradiation, but it makes it hard to compare titers to other species once you do this gamma irradiation.

In Paweska et al, the data is reported as a "percent positive" compared to a positive control their lab possesses. As a reader, without that positive control it is hard to say if those values are lower than expected. The neutralization titers are lower than we might expect; however, we do not have a frame of reference for what neutralization titers should be 20 days out for this pathogen. The authors themselves state "Neutralizing antibodies irrespective of the presence or absence of complement were rather low and not detected in all inoculated animals", but again what is "rather low"?

In summary, those are all relevant papers, we agree with your suggestion they should be included, but there were specific reasons we did not cite them.

L51: This line reads a bit as though you are infecting the bat with the guinea pig - suggestion to reword

R47: Thank you for the observation. We have rephrased the sentence as "*Since bats are the reservoir hosts of Nipah and Hendra, it remains unclear if bats generated weaker antibody responses than the species they were directly compared to (horses, guinea pigs, and cats) because bats generate poor antibody responses or because bats were being infected with a virus they have co-evolved with while the comparison species were infected with a virus they have not co-evolved with."* (Lines 40-47)

L55: It is not really true that these studies are predominantly focused on henipaviruses - you just did not cite the others

See R46:

L58-60: Epstein et al 2008 does not show great support for an association between poor body condition and high seroprevalence, nor does it test pathogen specific serum titers to my knowledge, only seroprevalence. Epstein et al 2020 actually finds a negative association with seropositive status and poor bat body condition using data from the same system

R48: In the Epstein paper, the bats with good body condition do not have SNTs greater than 80. They find the SNTs at 1:640 in the poor and fair bcs scores. Looking at the PvF stats, we agree with your point, but it's also why we wrote "*These observations suggest a complex interaction between food availability, nutritional status, and immune responses.*" What we see in the epidemiological literature are convincing correlations, but correlations that change their direction from paper to paper. This is one reason why we felt this work needed to be brought into the lab.

Table. ELISA and SNT results and univariate associations between serostatus and other variables for wild-caught *Pteropus giganteus* bats in India*

Characteristic	ELISA: No. NiV reactive/ no. tested	ELISA: No. HeV reactive/ no. tested	SNT: No. NiV positive/total (%) [median titer; range]	NiV SNT comparisons, p value†
Total	26/41	5/41	20/39‡ (51) [80; 5–640]	
Male	10/12	3/12	8/12 (67) [60; 20–640]	0.300
Female	16/29	2/29	12/27‡ (44) [80; 5–640]	
Lactating	12/20	2/20	8/19‡ (42) [80;20–640]	1.00
Nonlactating	4/9	0/9	4/8‡ (50) [80;5–80]	
Body condition score§				
Poor	5/9	0/9	1/9 (11) [640; NA]	P v F: 0.005;
Fair	16/24	5/24	16/23 (70) [80; 5–640]	F v G: 0.315;
Good	3/6	0/6	2/5 (40) [60; 40–80]	P v G: 0.505

*SNT, serum neutralization test; NiV, Nipah virus; HeV, Hendra virus; NA, not applicable; P, poor; F, fair; G, good.
†Fisher exact test p value significant at <0.05.
‡Two samples had insufficient plasma for SNT (both were ELISA negative); sample 1 was from a nonlactating adult female with a good body condition score (BCS) and the other was from a lactating adult with a fair BCS. A third sample, a nonlactating adult female with a good BCS had equivocal NiV/HeV SNT titers (*5*), which was attributed to an unspecified henipavirus and considered negative for NiV and HeV.
§Two pre-weaned pups (1 male, NiV SNT negative; 1 female, NiV SNT positive titer 80) were excluded from the BCS dataset because of their physical immaturity.

L63: As presented in the lines before, it is suggested that food shortages are associated with higher seroprevalence, so this alternative hypotheses that low food impairs Ab responses to drive viral transmission is confusing

R49: We have rephrased the paragraph:
In contrast to the weak antibody responses observed in experiments, field sampling of wild bats has occasionally identified individuals with robust serum antibodies[14,15]. While it is challenging to establish causality, bats' body condition or food availability often correlates with pathogen specific serum titers [15] , seroprevalence[8,14], or total serum IgG levels [16]. This correlation between food availability and serum antibodies is especially of interest because pathogen shedding and spillover events also correlate with food availability[17,18]. Counterintuitively, high resource provisioning in wildlife systems, which might be assumed to enhance fitness, often correlates with decreasing serum antibodies [16,19–23]. These observations suggest a complex interaction between food availability, nutritional status, and immune responses.

L76: sentence cutoff?

R50: Fixed, thank you.

L78: BALB/C - change "c" to lowercase as elsewhere in the paper

R51: Fixed, thank you.

L 80-81: What about experiment 1 and 3? You only summarize two of the 4 here. It would be helpful to have a summary for each and a corresponding hypothesis and result here.

R51: We have followed this suggestion. Line 76 now reads: "*Next, we challenged another group of JFBs with either the bat origin influenza A-like virus H18N11 or a replication incompetent Nipah virus VSV (Nipah-riVSV) after we had modified their diets.*"

L92: Either drop the s on bat or add an apostrophe to make possessive

R52: Thank you for the suggestion.

L97: These are the first mentions of NP-CGG and NP-Ficoll in the text. While I appreciate that PNAS asks for methods after results, you need to first tell us what these are before presenting results.

R53: We have edited the phrasing as "*In a pilot experiment, we immunized and boosted bats alone to optimize the inoculation timing. We immunized and boosted JFBs with a T-dependent (TD) antigen keyhole limpet hemocyanin (KLH) with Incomplete Freund's Adjuvant or a Tindependent (TI) antigen 4-Hydroxy-3-nitrophenylacetic conjugated to Ficoll (NP-Ficoll) with sterile saline (Supp. Fig. 1A).*" starting at line 83

L98: From the plot, this result appears to refer to boosting for NP-CGG but this should be clarified. Also, what test do these statistical tests result from? No methods are presented

R54: We agree the wording could be improved. The paragraph now reads:

To enable comparisons of mice and bats, we developed a competition ELISA for NP-CGG and NP-Ficoll (Fig.1G). Using the competition ELISA we found that post boosting (day 35 and day 56), NP-CGG immunized mouse serum displaced the B1-8 competitor antibody while bat serum did not displace the B1-8 competitor antibody (day 35: DF 1, F value = 61.65, P(>F) 4.99e-05; day 56: DF 1, F value = 9.871, P(>F) 0.0138). Prior to boosting (day 21), neither the NP-CGG immunized bat nor mouse serum displaced the competitor B1-8 antibody, indicating boosting is required to generate antibodies which compete with the B1-8 antibody (Fig.1E). Serum from NPFicoll (T.I.) immunized bats and mice could not displace the B1-8 antibody on days 21, 35, and 56, indicating SHM and affinity maturation are required to generate antibodies which compete with the B1-8 antibody (Fig.1F). Together, the results suggest an antibody response characterized by boosting, affinity maturation, and SHM can generate antibodies which displace the B1-8 antibody and that bats are less able to generate this antibody response compared to mice.

L102: Are these day 28 data shown for mouse? Plot only indicates day 35... and what happened on day 56 for Ficoll?

R55:

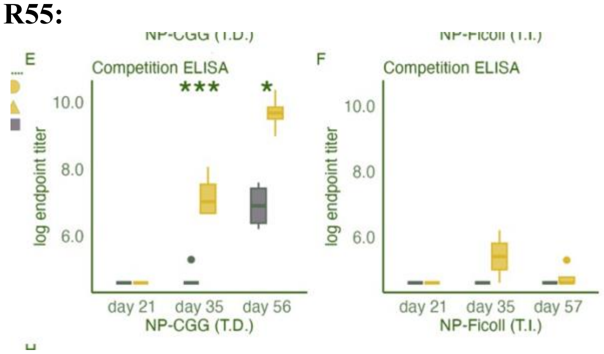

Day 56 data now included. NP-Ficoll doesn't lead to a boosting response, so at day 56 the animals all have very little serum antibodies present.

L115: Is mRNA an accurate measure of BCR diversity?

R56: We aimed to address the complexity and caveats surrounding this measurement in the discussion starting on line 181-191:

"Our findings provide a candidate mechanism behind the low avidity antibody responses observed in JFBs. By sequencing and comparing the BCR mRNA of bats and mice, we provided evidence that bats' BCR sequences have a higher alpha diversity than BALB/c mice post immunization and boosting, which corresponds to lower affinity antibodies. There are several possible mechanisms which could explain these results. Germinal center B cells undergo clonal bottlenecks as B cells with the highest affinity BCRs are instructed to stay in the germinal center, leading to a reduction in diversity. Second, late-stage germinal center B cells differentiate into plasma cells, which generate far more BCR mRNA per cell than non-plasma cells. Both processes could reduce the diversity metrics we observed in unsorted splenocytes, but more research will be needed to assess which processes are contributing. One major caveat of our findings is they came from only a single point in time. Germinal centers are dynamic processes and disentangling how bats' germinal centers mature over time will require the development of new methods and reagents specific for these bats"

L116: Need to specify that it is the JFB "BCRs" - not the JFBs themselves - that had increased sequence richness

R57: Fixed, thank you.

L119-120: Fig 2D vs Fig. 2d. Need to be consistent on how you reference figures

R58: Thank you. We have fixed the inconsistencies throughout the document.

L121: what does q=2 mean? See general comment #2

R59: See **R33**

L124: You reference the 'conceptual guide to measuring sequence diversity' here - seems a bit out of place?

R60: We have changed the citation to "Hill, M. O. Diversity and Evenness: A Unifying Notation and Its Consequences. *Ecology* **54**, 427–432 (1973)."

L126: "we sequenced BCR receptors" seems repetitive since the R stands for receptor

R61: Fixed, thank you.

L138: What does it mean that bats "had clear mesenteric lymph node responses based on enlarged MLNs"? Were bat lymph nodes were bigger after treatment in all cases? Or is this at baseline?

R62: We modified the language to communicate the findings as a qualitative observation. The manuscript now states on line 138: "*During necropsies, we harvested lymph nodes from mice and*

bats. We collected lymph nodes that were visually obvious and recorded the number of each lymph node type we harvested (Supp. Table 2).

L145: You need to reference Fig 3C here to point the author to the figure that shows this.

R63: We have restructured the paragraphs in lines 149-161 with more references to figures.

L151: This is a run-on sentence. Also, it would be easier to understand if described as "reduced neutralization capacity" or something to that effect rather than "improved viral entry"

R64: Done. See line 165: *"Surprisingly, by day 28, bats fed a protein supplemented diet had serum that reduced viral neutralization relative to day 0, although the mechanism behind this observation is unclear…"*

L156: You observed more IgHM sequence diversity in MLN vs what alternative...? Is this one treatment vs. the other?

R65: This statement is in comparison to spleen. We have also included sequencing read counts. We have modified the phrasing starting in line 182 for clarification:

"We sequenced BCR from bats 20 days post infection with H18N11 virus. Overall, we found less BCR sequencing reads in the mesenteric lymph nodes (MLN) than the spleen, with the notable exception of IgM sequences in the MLN of bats on the protein diet. (Fig.3C). Read counts were greater in infected bats than in controls. We also looked at diversity metrics via the Hill Diversity Curves. We found few differences by diet group. Again, the notable exception of the diversity of IgM sequences in the MLN where bats on the protein diet had higher diversity in the sequence richness ($q = 0$), Shannon-Hill diversity ($q = 1$), and Simpson-Hill diversity ($q = 2$) compared to the spleen (Fig.3E)."

L158: It would be easier to follow if you presented results from experiment 3 before experiment 4 in the same order as presented in the figure (experiment 3 in figure 3A vs. experiment 4 in figure 3B).

R66: We have followed the suggestion. Results now go in the following order:
Removing Dietary Protein Improved Neutralization of Nipah-riVSV *In Australia our lab has observed Pteropus bats switching from high protein pollen diet to a low protein diet during winter months, the season in which Hendra virus spillovers occur (unpublished data). Because of this diet switching, we tested the impact of a low protein diet on antibody production in response to NipahriVSV (Fig.2A). Bats fed a fruit-only diet developed a neutralizing titer as early as day 7 ($t = 4.74$, $p=0.005$, using a Bonferroni multiple test correction), while bats fed a protein supplemented diet never developed a neutralizing titer (Fig.2B). Surprisingly, by day 28, bats fed a protein supplemented diet had serum that reduced viral neutralization relative to day 0, although the mechanism behind this observation is unclear ($t = -9.47$, $p = 0.0002$).*

Removing Dietary Protein Improved Antibody Levels in Response to H18N11 Infection

To further investigate the effect of diet on antibody production in bats, we challenged bats with a bat-specific influenza A-like virus (H18N11) (Fig.3A). We further modified our experimental design

by placing bats on their diets for three weeks prior to viral infection. We also harvested RNA from mesenteric lymph nodes (MLN) and spleen tissue to sequence the BCR.

L159: There is no mention or discussion of Fig 3D - this should be explained or moved to the supplement (and explained there).

R67: We have decided to remove figure 3 as the information is also presented in supplemental figure 2C.

L164: Can you explain this proposed 'mechanistic insight'? I think you are suggesting that bat antibodies have lower affinity because BCRs are more diverse, but you should state this explicitly.

R68: We have modified the text (lines 201 to 210) to be more explicit.

L167: Rather stating that high affinity Abs "had a measurable effect" on MLN and splenic BCR mRNA diversity, state explicitly that they decreased it.

R69: Done. See lines 195-197: "*However, we also demonstrated that in response to either H18N11 or Nipah-riVSV, removing protein from the bats' diet resulted in higher serum titers and decreased diversity of MLN and splenic B cell mRNA"*.

L170: Again, it is better to be specific - don't you mean this could explain the positive correlation between high seroprevalence and poor body condition observed in the wild?

R70: We have changed the language in lines 215 to 223, as follows:

"Our findings also suggest the diet of bats has a direct impact on their antibody response and can be manipulated to improve their antibody response. Antibody titers correlated with BCR sequence diversity from the MLN tissue in the food restriction experiment. Our findings shed light on the associations between body condition, food availability, and antibody metrics observed in wild bats. Previous work in bats demonstrated a connection between changing environmental conditions with pathogen shedding and zoonotic spillover. Our findings suggest that the food bats consume directly impacts their antibody response and offer a mechanistic connection between the quality of antibody responses in bats and ecological conditions. Considering the importance of maternal antibodies to confer passive immunity to pups, it is possible that the effects of diet consumed by pregnant female bats could influence the levels of immunity passed to the offspring, influencing viral dynamics."
L180: Again, be specific- do you mean that high Ab titers correlate with low BCR sequence diversity?

R71: We have changed the language in lines 215 to 223.

L187: This feel pretty speculative. What is the hypothesis that you are suggesting?

R72: Agreed. It was speculative and we decided to remove it.

L192: First, you need to italicize Myotis. You also need to state that the paper referenced was a study on Myotis lucifugus bats

R73: Done. Section in lines 202-205 now reads as: "*SHM is deemed critical for generating highaffinity B cells in mammals. However, the presence and extent of SHM in bats has not been directly assessed, and subsequent studies in other bat species have not corroborated the study that suggested that Myotis lucifugus bats have an unusually high number of variable heavy chain genes.*"

L195: As worded, it almost sounds like the authors are suggesting their findings run contrary to a hypothesis of reduced SHM in bats, while I interpret it to be to the contrary. I think the authors mean to say that it is "important to note that our analysis does not provide direct evidence that bats undergo limited SHM, though it does support prior work indicating higher BCR diversity and lower affinity antibodies in some bat species". From this paper, it seems like healthy bats (receiving sufficient protein) might have reduced SHM (though I recognize this is not directly tested) but that bats on low nutrient diets might induce SHM more robustly.

R74: We consider plausible that the rate of SHM is comparable between bats and other species and the observed diversity/affinity phenotype might be explained by selection to proliferate/differentiate in the germinal centers. We have changed the language in the discussion to emphasize this possibility.

L205: I think the suggestion of bats as hosts of more zoonoses is restricted to viruses (not pathogens broadly) in Olival et al. 2017, Mollentze and Streicker 2022 contradict this, so it is an incomplete statement as presented. I would also suggest moving the group of citations here (41-44) to be scattered throughout the sentence, as the other papers references refer to earlier parts of the complex sentence.

R75: We have modified the phrasing in lines 222-224 as follows:

Bats can live in large, dense roost structures with numerous co-roosting species which enable transmission between species[49,50]. Bats live especially long lives for their body size[51], and it has been suggested that bats host more zoonotic viruses than any other mammalian clade[52]. Therefore, it may be advantageous for bats to invest in broadly neutralizing antibodies.

L226: This should say humoral "immune" response?

R76: Correct. Fixed.

L233: To connect the experiments with the lab methods, it would be helpful to say that blood was collected for downstream ELISAs to measure antibody responses to T-dependent and -independent antigens. In the methods, it says these bats are immunized but does not explain why

R77: Done. See lines 281-286:

"KLH had a target volume of 50 µL per bat at a concentration of 10 µg / 50 µL. The NP-Ficoll immunogen had a target volume of 50 µL per bat at a concentration of 0.5 µg NP/µL. Animals were immunized on day 0 and boosted on days 21 and 42. ***Blood was collected for downstream ELISAS to measure the antibody response to TD and TI antigens.*** *Blood was collected on days 0, 7, 14, 21, 28, 35, and 42 via the cephalic vein. Animals were euthanized on day 45. All bats were fed a diet of fruit (e.g., honeydew, cantaloupe, watermelon, bananas, strawberries, oranges) with a*

supplement of ground Mazuri Softbill Protein powder (Mazuri Exotic Animal Nutrition SKU 0053414).”

L243-244: The line reads that the pregnant bat was immunized IP to avoid fetal harm... but all the bats were immunized IP. Did you mean to write something else?

R78: Thank you for noticing this error, we have fixed it. The individual was immunized subcutaneously.

L264: drop s in "reservoirs" - typo

R79:

L266: missing period

R80:

L268: were bats on these diets for two weeks in both experiment 3 and 4? It does not say for experiment 3

R81: We have included graphical summaries as follows:

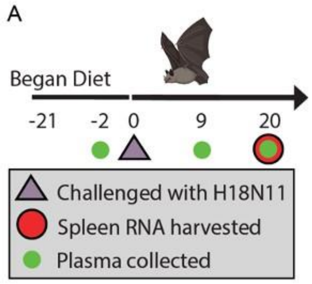

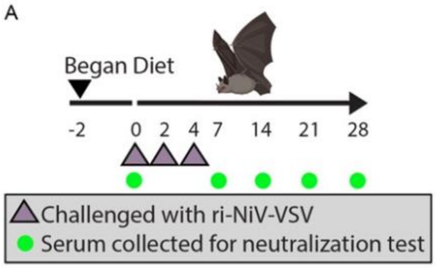

L270 : Bats were euthanized prior to spleen harvest right? Say this. What happened to the other bats?

R82: All bats were euthanized, and spleens were only harvested from dead animals. See lines 343 -326: “*After euthanizing all animals, spleens and mesenteric lymph nodes were harvested for RNA. Three individuals per infection group were selected randomly for BCR sequencing. One negative control animal from each diet (non-infected) was also selected for BCR sequencing.”*
L271: How many controls vs. infections were made? You only say that 8 bats each were assigned to the two diet groups but it is not clear how many were infected vs. not. Were there non-challenge controls in experiment 3 as well?

R83: We have included details in lines 321:
“*Bats were placed on their diets for three weeks. After three weeks, six bats in each group were inoculated intranasally with 5 x 10^5 TCID50 of H18N11 virus. Two bats per diet group were not*

infected and kept in a separate room. Bats were kept on their dietary regimes throughout the infection."

L280: Why is the VNT listed before the competition ELISA in the Methods when this was the last experiment It would be easier to follow if listed chronologically

R84: The VNT is now included after the other ELISA, see lines 316-317.

L479: Experiments is why capitalized... is this another typo?

R85: Fixed.

L481: Didn't you also measure F4 mAB in experiment 4 via indirect ELISA? This is not listed here

R86: Text modified on line 521-523: We serially diluted the serum into NAP buffer, added 100 µL to each well, and incubated the plate for two hours RT. We washed the plates three times in PBST and probed using protein G:HRP, goat anti-IgG-Heavy chain HRP antibody (Southern Biotech Cat. No.: 1031-05), or our own monoclonal antibody F4.

L501: The sentence is incomprehensible - are there words missing?

R87: Fixed. Words were accidentally cut out during the final round of editing.

Figure specific comments:

R88: We have updated figures for clarity and incorporated most of these suggestions in the figures, or added additional information to figure legends when as needed.

Fig1B - plot is fuzzy - what are the dots above and below the boxplots? Are these outliers? They are hard to see. Also, what is this the endpoint titer for? The axis should specify

R89: We have included details in the figure legends and in the figures themselves. Outliers are easier to visualize now.

Fig 1D - This could be clearer. Some words might help. Also, shouldn't it be shown for both NP-CGG and NP-Ficoll?

R88: Done. The figure displays both antigens side-by-side.

Fig 1E - It would be helpful to give these two plots different letters to better explain their differences in the caption - or you could facet them by NP-type

R89: Changed, please see R32

Fig1B/1E - It would be helpful to show where the boosting took place on these plots

R90: We agree, but including this information does make the figure challenging to read. Would the following be an acceptable improvement?

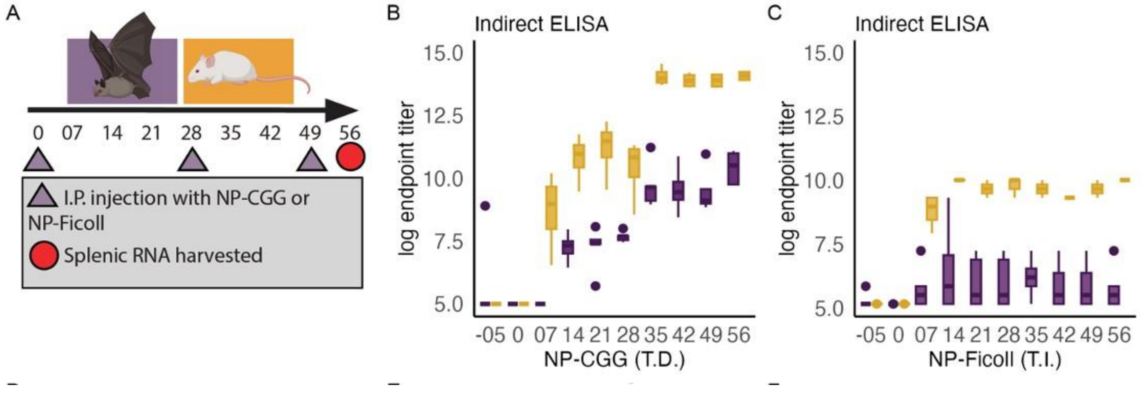

Fig2 B - what does the "contains VDJ junction" bit refer to? This is not explained in the caption

R91: The figure caption for supplemental figure 2A has new text that we hope makes this more clear. See line ~706

Fig2 C - This is blurry. Also definitely do not need the models written out here in the main figure, plus the text is too small to read

R92: Removed

Fig 2D - what is the x axis?

R93: See R33

Fig3A - it would be helful to include the statistical outputs here in the caption as well as the main text

R94: Lines 162-165: Main text*: Because of this diet switching, we tested the impact of a low protein diet on antibody production in response to Nipah-riVSV (Fig.2A). Bats fed a fruit-only diet developed a neutralizing titer as early as day 7 (t = 4.74, p=0.005, using a Bonferroni multiple test correction), while bats fed a protein supplemented diet never developed a neutralizing titer (Fig.2B).*

Figure caption*: Figure 2:* *Experiment 3; Diet Manipulation Impact on Bat Humoral Immune Response to Nipah-riVSV Challenge A. Timeline of the experiment B. Serum neutralization of Nipah-riVSV entry. Data is normalized to day 0. Asterisks indicate significant difference compared to day 0 values, within a diet. Results are shown with serum at a 1:30 dilution. Boxplots show the 25%, 50%, 75% percentiles, lines indicate the smallest and largest values within 1.5 times the interquartile range, dots indicate values beyond that. P values refer to Student's t-test ('*' P <0.005 for multiple test correction)*

Fig 3B - looks squished. Also, I believe the G-HRP mentioned in the caption the same thing as the "antibat IgG" in the figure but it would help a lot to use consistent terms in both places and in the methods

R95: Language now standardized throughout document.

Fig 3D - This could be supplemental

R96: Since it has the same optimization procedure, data, and cutoff value as the other experiments, we've removed it.

Fig 3E - As mentioned in the general point #2, these graphs need explaining

R97: Agreed, see R33

SFig1 B - It would be helpful to specify which of these is T dependent and independent. I have to keep going back to the text.

R98: This is a helpful suggestion and has been incorporated.

SFig2A - It would help to color the proteins differently in addition to showing as different shapes

R99: See R100, plots removed.

SFig2B - I'd recommend faceting this plot pre- and post boosting rather than repeating the y-axis twice. Also this way, you could have strip labels to label which panel is pre and vs. post boosting which would make it easier to read. For the post-boosting panel, this should specify that the x-axis is 'days since initial NP-CGG immunization'

R100: We've removed these plots. For context, the IC50 assay required months of optimizing, but in retrospect do not provide additional information. The assay has limitations, which is why the figure is split into two graphs. Pre and post boosting, we concluded from optimizing the assay that the IC50 results are not comparable pre and post boosting, so we split the results into two graphs.

SFig2C - These results are not discussed in the text. How am I supposed to interpret this?

R101: We removed the results and presented only the indirect ELISA.

Supp Table 1: Helpful! I learned about the controls here. Looks like no controls in experiment 3 - is this because it was simultaneous with experiment 4?

R102: We did not have non-infected bats in the NiV-riVSV infection study. The inferences were intended to be made between the two diet groups. Once we observed the changes, we ran experiment 4. We included non-infection controls in experiment 4 because we were interested in the effects of the diet alone. We chose not to pursue that story here.

Supp Table 2: This is messy, with headers cut off in several cases. Does "LN" mean "lymph node"? What are the units of the values?

R103: We have updated the table and changed how it was imported into word.
