# Supplementary material for: Bats generate lower affinity but higher diversity antibody responses than those of mice, but pathogen-binding capacity increases if protein is restricted in their diet
Source: PLoS Biol. 2024 Sep 24;22(9):e3002800. doi: 10.1371/journal.pbio.3002800 (PMC11421821; doi:10.1371/journal.pbio.3002800)
Supplement: Attachment — Submitted filename: plos_biology_reviewer_comments_5_22.docx [file pbio.3002800.s009.docx]

Reviewer #1:

After reviewing the latest manuscript and considering two previous rounds of reviews, my overall opinion is that this paper presents highly intriguing results that could be suitable for a short paper. I concur with the earlier reviews that this is manuscript covers a substantial amount of work and commendable efforts to address the inherent difficulties that arise with comparative immunology in non-model species.

Were the authors to consider shortening this manuscript, this could be achieved by focussing on the main strength of the paper, which I believe are the challenge experiments under dietary manipulation (Figs 2, 3 and associated text). I fully support the need for a comparative approach and commend the efforts made to address biases caused by species-specific reagents, specifically, the competition ELISA. In addition, I believe the diversity metrics are interesting even if it isn't yet clear how they relate However I have serious concerns about other elements of this comparison.

1. The choice of the BALB/c mouse as representative of "other mammals" (L69) as serious limitations. Indeed, unlike the Jamaican Fruit Bats used in this study, BALB/c mice are inbred, and thus homozygous at each allele, including the MHC. While generation of BCR diversity is only partly dependent on the genetic background, the lower BCR diversity in mice reported in figure 1H therefore could simply be a consequence of not having used an outbred mouse. As a consequence, statements like "Overall, JFBs generated a weaker antibody response and possessed more BCR mRNA diversity compared to mice" (L24-25) should probably be qualified, since these differences may be due to the study design rather than to biological characteristics of bats. The paper would be more compelling if the authors could replicate the BCR diversity study in outbred Mus musculus.

R1: We agree that outbred mice would be a better comparison and agree that we should qualify our statement on the comparison to specify BALB/c mice. We have expanded the discussion by adding paragraphs that address these concerns (see below, lines 244-251). We added citations that have guided our thinking on comparative immunology work and the limitations of standard mouse models.

We will investigate the use of outbred mice (such as pathogen naturalized mice) as a comparison species for future studies. Establishing the colony of bats and preparing for the experiments described in the paper took over a year, and we anticipate that establishing a colony of another non-model species would require similar effort.

Lines 244-251: *“Furthermore, as we begin to manipulate the diet and housing conditions of bats, we also need to consider the housing conditions, and strains, of mice. In our study, we used the inbred BALB/c mouse strain. However, incorporating outbred mouse strains could improve the study by better representing the genetic diversity found in natural populations. We should also consider using pathogen naturalized mice in future studies (mice which have encountered various pathogens in their environment ^45^.) These mice have different circulating lymphocyte populations and it would be useful to assess if mice with prior microbial exposure change their B-cell receptor diversity following immunization. The incorporation of more naturalized mice in future comparative immunology studies involving bats could potentially yield more relevant and insightful results.”*

2. It isn't clear how differences in BCR diversity impact antibody affinity. In particular, the claim that "affinity maturation process is reduced in Myotis bats due to an expanded antibody variable region gene repertoire [...]" (L63) is highly speculative, and would require experimental validation or further support from the literature. Of the citations given (Bratsch 2011, Buter 2011, Shaw 2012), only Bratsch mentions this idea but as far as I can tell, merely as a hypothesis. In fact, a diverse initial repertoire generated by V(D)J recombination can be beneficial because it increases the likelihood that at least some BCRs will have an initial, albeit low, affinity for a given antigen, providing a better starting point for subsequent affinity maturation. The authors should consider dropping this claim if a trade-off between affinity maturation and antibody variable gene repertoire cannot be better supported.

R2: We agree with the reviewer and appreciate the reviewer’s attention to detail in those citations. We have now made it clearer that the downstream effects of the expanded V region put forth in Bratsch 2011 is a hypothesis. Our conclusion that the evidence behind this idea was lacking was a motivator for this project in the first place.

We clarified our wording regarding this evidence and reworked several other sections to emphasize that our study does not directly measure or assess affinity maturation, somatic hypermutation, nor the Vh germline diversity mentioned in Bratsch 2011. Instead, our diversity metric is referring to quantifying B cell clonal diversity. We edited discussion paragraph three to make it clear our results do not support, or contradict, Bratsch 2011.

There are several possible mechanisms which could explain why higher clonal diversity is associated with low affinity serum antibodies. We have made these potential mechanisms clearer.

Lines 202-205:

*“Our BCR sequencing data provides a candidate mechanism behind the low avidity antibody responses observed in JFBs that can be further explored in subsequent studies. By grouping BCR mRNA sequences of bats and mice into clones, we provide evidence that bats’ B cell response is characterized by higher alpha diversity than BALB/c mice post immunization and boosting. There are several possible mechanisms which could explain the association of higher B cell clonal diversity and low affinity serum antibodies. Germinal center B cells undergo clonal bottlenecks when only high affinity B cells are instructed to stay in the germinal center, leading to a reduction in diversity^40^. Second, late-stage germinal center B cells differentiate into plasma cells^48^, which generate far more BCR mRNA per cell than non-plasma cells^49^. If bat germinal centers are more permissive, enabling low affinity B cells to continue proliferating, or if plasma cell differentiation rates are lower in bats, this could result in the reduced B cell clonal diversity and contribute to low avidity serum antibodies. However, more research will be needed to assess which processes are contributing. One important limitation is our sequences came from a single point in time and from unsorted splenocytes because there is a lack of reagents for sorting bat lymphocytes. B cell training and selection within germinal centers is a dynamic process, and disentangling how bats’ germinal centers mature over time will require the development of new methods and reagents specific for flow cytometry in bats.*

*Our study cannot be used to make inferences into the rates of somatic hypermutation (SHM) in bats. SHM is critical for generating high-affinity B cells in mammals. A previous publication hypothesized some bat species’ B cells may undergo SHM at rates lower than observed in model species because they possess an expanded number of V heavy chain (V_H_). This in turn enables these bat species to generate a robust B cell germline repertoire from just VDJ rearrangement, hypothetically. However, other bat species appear to have a similar number of germline V_H_ genes as humans^50,51^ . Our study did not assess rates of SHM nor do our BCR mRNA diversity metrics provide reliable evidence of our bats’ germline repertoire. Considering that SHM is evolutionarily conserved and is found in basal vertebrates^52^, we suspect it is unlikely that bats have lost this process. Indeed, JFBs in our study and others have been shown to express AID^53^, a critical enzyme for SHM. While SHM is likely critical for the proliferation and differentiation of B cells, without an annotated immunoglobulin heavy chain variable gene locus for JFBs, the data from our study cannot be used to make inferences into the rate of SHM in these bats.”*

3. As a consequence of the previous point, it isn't clear how the JFB vs BALB/c comparison links up with the section on  the effects of dietary protein, (experiments 3 & 4).

R3: We have clarified why we want these experiments tied together in the text. We have modified four of the five paragraphs in the introduction (lines 50-88) to clarify why these studies are related. We have also modified the topic sentences (lines 86, 103, 114, 145, & 158) in the results section which I hope makes the connection between these experiments clearer.

Lines 50-88:

*“When bats have been infected alongside other mammals, bats have generated the weaker antibody responses ^11–13^ . However, these comparative studies used viruses that are coevolved with the experimental bat species, so the weaker response seen in bats could be due to coevolutionary history with the virus. Single-species studies that have infected or immunized bats resulted in lower serum titers than researchers expected^14–16^, however without a comparison species it is difficult to predict what antibody response should have been expected. It could be more feasible to draw conclusions about bats’ antibody responses if bats were immunized alongside model organisms using well studied antigens, such as T-dependent antigens which can have well characterized responses in lab mice^17–21^.*

*There are few proposed mechanisms in bats which explain their purported low antibody:antigen bond strength (i.e., antibody affinity). Antibody affinity typically increases after exposure to a foreign antigen. This process, known as affinity maturation, is mediated by the B cell specific mechanism somatic hypermutation (SHM) and can be induced following infection and immunization with T-dependent antigens. Previous research hypothesized that the affinity maturation process is reduced in Myotis bats because the bats may have an expanded antibody variable region gene repertoire, limiting the need for SHM to generate a sufficient antibody repertoire. Subsequent studies found that following infection, Artibeus bats did not increase the expression of AID (activation induced cytidine deaminase), a critical enzyme which is needed for SHM ^22^. While either mechanism could reduce affinity maturation and lower antibody affinity, the evidence behind these claims is weak.*

*In contrast to the weak antibody responses observed in experiments, field sampling has occasionally identified wild bats with high titers of serum antibodies^23,24^. While it is challenging to establish causality in ecological settings, bats’ body condition or food availability has been observed to correlate with pathogen specific serum titers^24^, seroprevalence^8,23^, or total serum antibody (IgG) levels^25^. Furthermore, in some bat spillover systems, food availability is thought to be a key driver of pathogen shedding and spillover^26,27^, but an immunological mechanism linking the two has not yet been established. Any prediction as to how antibodies might be impacted by food shortages is complex, as resource provisioning in wildlife systems, which might be assumed to enhance fitness, can correlate with decreasing serum antibodies^25,28–32^. Interestingly, experimental studies in mice have shown that manipulating metabolic pathways changes antibody affinity and germinal center dynamics, the primary location of SHM for B cells ^33–35^. While many of these experiments relied on antibiotics or gene knockout mice to modify metabolic pathways, new work has demonstrated diet manipulation alone changes the B cell receptor repertoire diversity in mice^36^. Together, these results suggest a complex interaction between food availability, nutritional status, and the antibody responses.*

*To directly test whether bats generate lower affinity antibodies than mice and to determine if diet may impact the affinity of their antibodies, we performed a series of three experiments on Jamaican fruit bats (Artibeus jamaicensis; JFB). First we immunized JFB bats alongside BALB/c mice with commonly used T-dependent or T-independent immunogens to measure their antibody response to a non-infectious challenge. T-dependent immunogens elicit SHM and affinity maturation whereas T-independent immunogens result in an antibody response, but do not require T cell help and do not elicit SHM or affinity maturation. We hypothesized that bats would generate lower affinity antibodies than mice in response to T-dependent antigens but would generate comparable affinity antibodies to mice with a T cell-independent antigen. We next assessed how sensitive bats’ antibody responses were to changes in their diet. We started by removing the protein supplement from the standard JFB diet, then challenging the bats with a replication incompetent Nipah virus VSV (Nipah-riVSV). We then progressed to a viral infection study using the bat origin influenza A-like virus H18N11^37,38^ . We hypothesized that removing the protein supplement from their diet would have a deleterious effect on their antibody response.”*

Based on the issues mentioned above, I would suggest completely removing the mouse comparisons from this paper, and better justifying the causal link or at least the complementarity between BCR diversity and antibody affinity. Additionally, there are a few minor points that should be addressed.

R4: We recognize the reviewers point and have altered the text to show why these experiments belong together:

In brief, the mouse/bat comparative immunology experiment provides supportive data and a (potential) mechanism behind the hypothesis that bats have lower affinity/avidity antibody responses than other mammals. This is an idea that has appeared in several review papers, but lacked evidence, in our opinion.

The diet work should be published alongside the comparative work because it shows that the B cell response is plastic and can be manipulated by dietary changes. These findings relate to our understanding of the ecology of wild bats, their roles as reservoir hosts, and the drivers of viral spillover. The results also show that diet may be a critical experimental condition for studies in bats, where there are not standardized protocols for feeding and housing these animals.

In summary, our work suggests that bats generated a ‘weaker’ antibody response than mice, but also that the magnitude of the bats’ antibody response is impacted by the conditions experienced by the bats (i.e. diet).

4. There should be better parity between the assays in experiments of Fig 2 (Nipah-riVSV) and Fig 3 (H18N11), especially analysis of the antibody subclasses and protective responses (neutralization or similar). Indeed, it is important to avoid readers assuming an antibody response is always protective.

R5: The work represents a progression from a study with Nipah-VSV, followed by a more comprehensive study for which we had to develop new reagents, such as the anti-light chain assay, that were not available for the original experiment. Neutralization assays, such as the one established for the Nipah-VSV experiment, are challenging to establish for the H18N11 Influenza virus.

On line 171-172 we’ve now clarified we had no neutralization assay for the H18N11 result: *“Again, counter to our original hypothesis, at the terminal time point (day 20 post infection) the bats on the fruit-only diet had a stronger antibody response to the H18 antigen when we measured all antibody subclasses (i.e., IgG, IgA, IgM, etc.). Specifically, at the terminal time point the fruit-only diet had higher anti-H18 antibody levels when measured using the F4 monoclonal antibody (day 20: DF 1, F = 15.12, p = 0.003) (Fig. 3D). The F4 mAb recognizes a 25 kDa protein in reduced and denatured S-300 size fractionated black flying fox (Pteropus alecto) sera. 25kDa is the approximate size of the of Ig light chain (Fig. 3B). Thus, measurements taken using the F4 mAb do not differentiate antibody subclasses. Interestingly, we observed no difference between diet groups in anti-H18 antibody levels when measured using protein G, which binds mammalian IgG (day 20: DF = 1, F = 3.2, P(>F) = 0.10; day 9: DF = 1, F = 2.57, P(>F) = 0.14) (Fig. 3C). We did not have a neutralization assay established for the H18N11 virus and could not measure the antibodies’ neutralization capabilities.”*

*Line 159: After the neutralizing assay, no serum was remaining to use the F4 mAb antibody.*

3. The section "Macroscopic Findings of Lymphoid Tissue Responses" (L142-144) could be cut, as this has no straightforward implication for (protective) immunity. It might be sufficient to explain in the methods that enlarged LNs were extracted for further analysis.

R6: We thought it might be of interest for some readers after discussions at a recent conference about lymph nodes in bats (and how researchers rarely find them). However, given this feedback, we have removed this section.

**5. In support of the author's view, and in light of the observation that there was no difference in IgG concentrations in response to H18N11 (L157), it would be good to discuss the apparent lack of protection conferred by IgG.**

R7: In the fourth paragraph of the discussion, currently lines 243-251, we have added language to further address the IgG and H18N11 findings and put it in context of the current literature. We plan on testing a reinfection study in these bats to assess the protective capabilities of the IgG response.

*“While we found bats generated a relatively low avidity antibody response compared to mice, we also found bats' antibody responses could be improved by manipulating their diet. Specifically, restricting their dietary protein increased serum antibody levels to both a replication incompetent Nipah-G VSV and a live virus, H18N11 IAV. Although somewhat counterintuitively, similar findings to our study have been observed in wild animals, where, counterintuitively, improved access to food has been associated with decreased antibody levels^25,28–32^. In line with these findings, a meta-analysis of malnutrition in human children also shows complex and often surprising results^54^. Observational studies in children found that serum IgG levels are not depressed in mild or moderately malnourished children following vaccination and only decrease in severely malnourished children^54^. More surprisingly, while malnourished children have depressed levels of secreted IgA levels in mucosal surfaces, serum IgA levels increase^54^. Future work should determine which serum antibody subclasses in bats are impacted by dietary changes and if these differential antibody responses provide measurable differences in protection to reinfection.”*

6. Supplemental Figure 1 is confusing. The legend implies that the plots represent both bats and mice, but it appears that only bats are depicted in panels C and D.

R8: We removed the pilot study from the figure since it does not add substantially more information to the study. We also believe this change addresses questions about the methods changing between the pilot and experiment 2.

------------------------------------


Reviewer #2:

This review was completed by a PI and their postdoctoral fellow together with the permission of the editor. It reflects the feedback of both researchers. The paper by Crowley et al. presents some intriguing initial experiments on the humoral immune response of Jamaican fruit bats compared to the response of BALB/c mice, as well as the impact of diet on the humoral response. While preliminary, the results could be quite exciting. On balance, the discussion is well thought out, presents an interesting model for the adaptive significance of diverse but less neutralizing immunoglobulin populations, and full of appropriate caveats. However, we have several questions and concerns with the methodology and presentation of results that would clarify the strength of their results. If the major concerns and questions can be addressed and resolved, and the results still hold, it would likely make a great short report for PLoS Biology. We summarize our major concerns first and then suggest some minor, mostly text corrections.

R9: Thank you for taking the time to thoughtfully review our manuscript. We address your points below and throughout the paper.

Major revisions:

The way the manuscript is currently structured is somewhat disjointed and difficult to follow. It reads as four pilot experiments loosely grouped into two stories.

R9: We have modified both the introduction and the discussion to make it clearer how these experiments are connected and why we think it’s important to publish these experiments together.

We also modified the topic sentences in the results section to help readers follow the logic of our thinking as we progressed from experiment to experiment.

See R11 for additional details.

The results section can be hard to follow at times and reads more like a methods section in places - this could be remedied by including more rationale/justification/clarification for experimental decisions and the corresponding results.

R10: We restructured the results section to communicate our rationale and decision making more clearly for the series of experiment. We have edited the topic sentences (lines 86, 103, 114, 145, & 158) where we have implemented edits that address this concern:

*“We began by testing the hypothesis that bats would develop lower affinity antibodies than mice following immunization with a T.D. antigen but would have equivalent affinity antibodies to a T.I. antigen….”*

*…We began our comparison study by using an indirect ELISA, one of the standard techniques to assess antibody titers…*

*…To enable comparisons of mice and bats, we developed a competition ELISA for NP-CGG (TD) and NP-Ficoll (TI) (Fig. 1G)….*

*…We sequenced the B cell receptor (BCR) heavy chain mRNA in bat and mice splenocytes to assess the B cell clonal diversity. Our objective was to better understand why the immunized and boosted mice possessed higher affinity serum antibodies…*

*…We next tested the hypothesis that removal of dietary protein would have a detrimental impact on the antibody levels of bats. We tested this hypothesis for two reasons. First, food availability is linked to both pathogen shedding, but also antibody levels, in several bat spillover systems. In the Australian Hendra virus system, Pteropus bats have been observed switching from their preferred high protein pollen diet to cultivated low protein fruits during winter months^43^ , the season in which Hendra virus spillovers occur^44^ and starvation events in this system have been correlated with increased seroprevalence in anti-Hendra IgG antibodies^23^. Second, housing conditions have known impacts on the immune system of laboratory animals^45^ and we were interested in assessing the effect of removing the protein supplement from the bats’ diet…*

*…We next assessed if a similar effect of protein restriction results could be replicated with the bat-specific influenza A-like virus (H18N11) (Fig. 3A). We also started the bats on their diet for three weeks prior to viral infection to give the bats and period of adjustment to the diet prior to infection…*

*…We had observed in the comparative immunology experiment that bats had both lower affinity antibodies and higher BCR diversity. Thus, we were interested in assessing if the higher titers observed in the fruit-only diet would be associated with similar changes in BCR diversity…”*

The first story compares immune responses in bats and mice to non-infectious immune challenges. The second story looks at the effect of diet on antibody responses in bats to pathogens. Each experiment was conducted differently, with variation in the antigens used for challenges, challenge timings, experimental procedures (e.g. inoculation route; ELISA vs neutralization assays), analyses (some have BCR analyses and others do not), and study populations (all male; all female). This makes it quite hard to compare across studies - indeed are the studies comparable? -- and uncover general themes R11a. The experiments are each presented separately in the results which exacerbates the piecemeal nature of the manuscript R11b. In particular, the pilot study was conducted with different reagents and different timepoints, and it's unclear why they included this information in the manuscript. Can the authors provide more rationale for the pilot study and relate it better to the following studies? R11c None of the results are directly comparable but perhaps the results could at least explicitly group lines of evidence that support similar hypotheses, e.g. there may be increased titers of antibodies in fruit-only groups R11d?

R11a-b: We appreciate the thoroughness of the reviewers and recognize that there were some limitations of the experimental design that was outside of our control.

You are correct that methods and sex changed. For a non-model species with limited individuals available, we were not always able to get the sex we requested. We preferred to use only males, but there were not enough available at the times we needed them.

Per experimental procedures, we developed several assays used in the paper while we were conducting the experiments. For example, we did not isolate the F4 hybridoma until the H18N11 experiment had ended. We had no remaining serum from the Nipah-VSV experiment, so we could not test those bats using the F4 hybridoma antibodies. For the Nipah-VSV, the Aguilar lab at Cornell had a well-established neutralization assay. However, these assays are challenging to establish and we have not successfully developed one for the H18N11 virus . While this does make the experiments hard to compare, it is also a strength that we observed similar findings with different experimental procedures and reflects some of the challenges working with non-model species.

R11c: We have removed the pilot. From the pilot we saw that the bat IgG antibody response, pre boosting, were barely measurable. We thought this could be interesting to a reader. However, we realized without a comparison species it was challenging to draw too many conclusions. Thus, we repeated the experiment, but a) included mice and b) changed the assay conditions to use the NP-Ficoll and NP-CGG (enabling a more direct T dependent and T independent experiment).

R11d: We have modified parts of the discussion section which we believe better groups sets of results together.

We would also like additional information to evaluate the validity of their results, specifically their calculations of BCR diversity. How are read counts quantified? What's the pipeline/normalization procedure? How are the authors accounting for variability in sequencing between samples?

This is information that should be in the results section because it has huge impacts on the trustworthiness of the results. A visual inspection of figure 3c (the only place read depth is supplied) suggests a very strong correlation between sequencing depth and inferred diversity. Also why were the uninfected control data not plotted? The authors state that they used bootstrapping to create 95% confidence intervals and determine differences in diversity but don't explain how their bootstrapping was done. Looking in the documentation for Change-O it merely states it uses bootstrapping but many R packages that do bootstrapping do so by resampling with replacement to the original sample size. In this way the read depth could dramatically skew the results. The authors should subsample each individual down to the lowest read count of any sample and rerun their analyses, as well as examine how their inferred diversity metrics correlate with the read depth from each individual to ensure this is not an artifact. The authors also need to report their read counts and normalization for all BCR analyses, not just the flu infection experiment.

R12: Thank you for these helpful comments. We added more text to explain the methods regarding sample size (and/or read depth) impacts on diversity metrics. We have modified the text to include more details on our bioinformatics pipeline and the rarefaction process for clones.

Regarding the normalization to address read depth, differential sampling depth is accounted for in the R package we are using by employing a Chao1 rarefaction process in the Hill Diversity curves. Thus, data were rarefied, Hill Diversity was calculated, then those data were bootstrapped (with replacement) to generate the confidence intervals.

To verify that the R package we are using (Alakazam package (version 1.2.1)) was indeed accounting for read counts and to assess the influence of differences in read counts, we performed a simulation experiment in R where we could manipulate the sample sizes, species distributions, and clone numbers from two individuals. In a scenario where one individual had far more read counts than another sample, the Hill diversity curve output using the Alakazam package remained unbiased, as the authors of the Alakazam package had suggested it would be. We've included the R code that we initially used for this simulation. Here, we can modify the read depth in two populations with similar underlying population distributions, and the rarefaction process in alakazam results in Hill diversity curves that are indistinguishable. Thus, we feel that using this method is robust to variation in read depth; but, for the samples with very low depth, we added language to highlight that the estimates, in these specific circumstances, need to be interpreted with caution.

We only observed instances with low sequencing depth in the diet manipulation experiment. Here, we obtained very few reads from the mesenteric lymph nodes from any bats in the fruit-only group. From our understanding of the Hill diversity curves with the Chao1 rarefaction, the estimates can become unreliable when sample sizes are small. Our goal in including the read counts was to be transparent, so we included the sequence counts in Fig. 3C. However, we should have been explicit that due to the low read counts in that group, the rarefaction process, and thus the Hill diversity estimates, need to be interpreted with caution for that section. For these reliability reasons we did not include the control animals in the Hill diversity counts because, again, we obtained so few reads from these infected animals. We agree this should be clear to the reader and have modified the text accordingly in the figure 3 caption.

samples = 30

clone_num=1000

clones = paste0("clone_", seq(1, clone_num))

bias_factor = 10

cond1=LaplacesDemon::rdirichlet(1, rep(1, clone_num))

dat = sample(x = clones, size = samples, replace = TRUE, prob = cond1)

dat <- as.data.frame(dat)

dat$clone_id = dat$dat

dat$consensus_count = 1

dat$group = "A"

dat.2 = sample(x = clones, size = samples*bias_factor,replace = TRUE, prob = cond1)

dat.2 <- as.data.frame(dat.2)

dat.2$clone_id = dat.2$dat.2

dat.2$consensus_count = 1

dat.2$group = "B"

dat.j = bind_rows(dat, dat.2)

unique(dat$clone_id) %>% length()

unique(dat.2$clone_id) %>% length()

x <- alakazam::alphaDiversity(data = dat.j, group = "group", clone="clone_id", copy = "consensus_count",min_q=0, max_q=4, step_q=0.1,

ci=0.95, nboot=100)

plot(x)

Line 173 to 186 now read:

***Steps*** *We processed, filtered, and analyzed the sequencing BCR reads using pRESTO and previously described germline free identification methods, as described in SI methods ^74,75^. Bat IgG and IgM sequences were separated using pRESTO by primers listed in Supp. Fig. 1A. Briefly, the TF-IDF (term frequency, inverse document frequency) quantified the distance between BCR sequences. We tested the number of nucleotides from the 3’ end of the BCR sequence to use in analyzing sequences (Fig. 2B). We used hierarchical Bayesian mixture models in Rstan 2.21.8^76^ to bin sequences into cluster (“clones”). We assessed clonal diversity between groups using Hill Diversity Curves and Chao1 rarefication methods to account for individual variation in read depth, implemented using the Alakazam package (version 1.2.1) ^46,77–80^ (Fig. 2C). Statistical significance tests of the Hill Diversity Curves were done at the species level as described previously ^77^.*

*“****BCR Sequencing from H18N11 Infected Bats Yielded Few Reads and Diet Based Shifts in Diversity*** *We had observed in the comparative immunology experiment that mice had both higher affinity antibodies and lower BCR diversity. Thus, we were interested in assessing if the higher titers observed in the fruit-only diet would be associated with similar changes in BCR diversity. Because H18N11 was a G.I. pathogen, we collected abdominal draining mesenteric lymph nodes (MLN) mRNA in addition to splenic mRNA on day 20 post infection. Overall, we found fewer BCR sequencing reads in the mesenteric lymph nodes (MLN) than the spleen, with the notable exception of IgM sequences in the MLN of bats on the protein diet. (Fig. 3C). Read counts were greater in infected bats than in controls. We also examined the diversity metrics via the Hill Diversity Curves. We found few differences by diet group. Again, the notable exception of the diversity of IgM sequences in the MLN where bats on the protein diet had higher diversity in the sequence richness (q = 0), Shannon-Hill diversity (q = 1), and Simpson-Hill diversity (q = 2) compared to the spleen (Fig. 3E). We arrived at these conclusions from the separation of the 95% CI generating by bootstrapping. However, Hill diversity curves with Chao1 rarefication may have unreliable estimates when sample sizes are low or uneven ^46^, which is what we observed in this dataset, especially for lymph nodes samples harvested from bats on the fruit only diet (Fig. 3C) . Thus, these Hill diversity estimates should be interpreted with caution.”*

Lines **355-366 now read: “***We assessed clonal diversity between groups using Hill Diversity Curves and Chao1 rarefication methods to account for individual variation in read depth, implemented using the Alakazam package (version 1.2.1)”*

Last, this is the how figure 1E appears when you subsample in base R, then run the Hill Diversity curve function. However, if you agree, we will continue to use the version in the paper.

We found the presentation of the ELISA results for the mouse-bat comparison confusing. Do both ELISAs need to be in the main figure? If the indirect ELISA can't be used to compare the antibody responses of bats and mice then I believe it would be better included as a separate figure, where the authors highlight the implications this has on other studies (in the vein of their response to a previous reviewer comment "**We think this is an important point for readers, because we often see reports of 'low titers' in bats, but if this conclusion comes from an indirect ELISA, then this is a mistake**." We agree this is important to highlight but this point is currently obscured and should be plainly stated.)

R14: We agree that this is important. We also are concerned by presenting both indirect ELISA and competition ELISA future comparative work will continue to the use the former. However, we included both because we had few time-points on the competition ELISA our colleagues questioned the overall dynamics when we presented the competition ELISA alone. We have now just included the competition ELISA and explained that more time points are included in the supplementary figure 1.

Also, we wonder about the implications of their indirect ELISA results versus competition ELISA results especially in comparison to their pilot study. The authors highlight that in the pilot they did not see increased titers in response to the T dependent antigen until after boosting but the indirect ELISA results (even if comparisons between bats and mice are inappropriate, within species between time point comparisons should be valid) shows an increase in titers without boost.

R15: Based on the reviewer’s feedback, we removed the pilot. The pilot helped us design the mouse vs bat study, but the data is challenging to interpret so we agree it should be removed.

Why are the analyses done for the H18 and Nipah infections different? The authors went into much more detail for the H18 infection - can these same analyses on the BCR sequencing diversity be done for Nipah? If not why? If not, then they should consider reworking the results section to make Nipah a smaller part and more of a supporting piece of data for observations from H18.

R16: We ran the Nipah-VSV experiment first and wanted to repeat the experiment to see if the results would be observed again under different conditions.

Unfortunately, we did not collect splenic mRNA from the Nipah VSV bats so could not run BCR sequencing. We have noted that on lines 500-501: “*Splenic mRNA was not harvested from these bats and BCR sequencing could not be done.*” We agree that having the VSV work as a supporting part of the H18N11 study is worthwhile, however when presenting the work, we have found that the methods are easier to follow when the experiments are separated.

As for the serum analyses, for the Nipah-VSV a neutralization assay had been designed and optimized by the Aguilar lab at Cornell University. This assay required most of the serum volume available from the bats so we were not able to measure total anti-H18 antibodies (with anti-light chain hybridoma F4). For the H18 experiment, we did not have a neutralization assay established. Fortunately, by the time we finished the H18 experiment we had successfully isolated and grown the anti-light chain hybridoma F4 and were therefore able to measure titers but not neutralization. We added text to clarify these differences.

Lines 123-141, Figure 1H,I: In this study the authors used BALB/c mice as a comparison. BALB/c is a highly inbred lab strain of mouse that is known to be much more germline-focused, with less nucleotide addition in the CDR3 region than the human repertoire. Additionally, its V gene repertoire and immunoglobulin repertoire in general is very distinct even from C57BL/6, another inbred strain of Mus musculus (Collins et al. 2015, Phil Trans B, <https://doi.org/10.1098/rstb.2014.0236>).  This known impact of inbreeding with likely lower diversity BCR repertoires would seem to bias the comparison between (relatively outbred) bats and a highly inbred, homozygous mouse line. In any case, the authors need to address how their choice of mouse impacts their comparison.

R17: Thank you for raising this important point. We have added two paragraphs to the discussion text to discuss the limitations of our mouse models and comparative immunology models more generally:

*“It remains uncertain if further modifications to diet and housing conditions can improve serum antibody titers in bats. Countless housing conditions could impact immunological assays, and while establishing physiologically relevant housing conditions is important, it is unappreciatively complex. For instance, incorporating cages that enable bats to fly more freely, or providing diets that better mimic their wild food sources, could also have impacts on their antibody titers. These questions underscore the complexity of comparative immunology work.*

*Furthermore, as we begin to manipulate the housing conditions of bats, we also need to consider the housing conditions, and strains, of mice. In our study, we used the inbred BALB/c mouse strain. However, incorporating outbred mouse strains could improve the study by better representing the genetic diversity found in natural populations. We should also consider using pathogen naturalized mice in future studies, mice which have encountered various pathogens in their environment ^40^. These mice have different circulating lymphocyte populations and it would be useful to assess if mice with prior microbial exposure changes their B-cell receptor diversity following immunization. The incorporation of more naturalized mice in future comparative immunology studies involving bats could potentially yield more accurate and insightful results.”*

Line 174/ Figure 1E and F: Are the responses really "weaker" when compared to mice? They've shown more diversity in sequences even though there is less binding in a competition assay. What about straight B cell/antibody number? The response may be less specific, but not "weak" in terms of response rate/volume/activation. I have issues with the word "weaker" here. It could also be equally strong just not binding the exact same epitope that the competitor antibody binds and therefore not excluding it. Similarly, why were the competition assay results only done at three time points versus the 10 timepoints at which the indirect ELISA was performed?

R18: We changed the wording to “lower avidity” on the first line of the discussion and in the abstract. We think that is most accurate since we do not the know the class of the antibody in these results.

Per antibody counts: we attempted in the past to get accurate counts of antibodies in serum. However, this was a technically daunting pursuit and we did not feel confident with the methods or our subsequent results.

Per counting B cells: at the time of the experiment, we lacked basic antibodies for flow cytometry. As a workaround, we conjugated a fluorophore to our antigens. We thought this antigen could be bound by the BCR and we could sort antigen specific B cells. This process worked well for mice but failed for bats. We speculated that this was because the BCRs lacked enough affinity to quickly bind the fluorophore labeled antigen. We are interested in trying this again and incubating the bat lymphocytes with the labeled antigen for longer periods of time and seeing if this helps.

We only realized the indirect ELISA were biased after running a series of tests. By this point, we did not have sufficient serum to run the competition ELISA on all time points. JFBs are approximately 30g and blood draws are more difficult that in BALB/C (in our experience). We added a sentence clarifying this on line 371: “*Unfortunately, sufficient serum was not available to test each time point where blood was drawn.*”

How do the T-dependent and T-independent responses change with diet? Why was this experiment not done? Can the authors better link the initial mouse vs. bat study to the later diet studies?

R19: The reviewers have pointed out a critical question that we are eager to address. See R17 for the language we added to this.

Briefly, we want to modify the bats diets and immunize them with NP-CGG simultaneously with mice. However, if we run this experiment, we also want to test how housing conditions impact the antibody response of mice. This opens a larger set of questions about comparative immunology: how do we differentiate results caused by the biology of the animal versus results caused by the housing conditions? When we compare two species, are we comparing their immune systems or are we comparing how their immune systems perform under unnatural housing conditions? We added another paragraph to highlight this point and would like to test some of these questions in the future.

The bioinformatics section of the methods needs to be expanded. The authors need to include program names and versions for all steps in the processing pipeline. In many places they state that things were done "as described previously," but there needs to be more explanation with a mention of the tools used and basic processing pipeline (which seems to be the immcantation portal?).

R20: Thank you for this suggestion. We have changed the Bioinformatics steps to now state:

*“****Bioinformatics Steps*** *We processed, filtered, and analyzed the sequencing BCR reads using pRESTO and previously described germline free identification methods, as described in SI methods ^68,69^. Bat IgG and IgM sequences were separated using pRESTO by primers listed in Supp. Fig. 1A. Briefly, the TF-IDF (term frequency, inverse document frequency) quantified the distance between BCR sequences. We tested the number of nucleotides from the 3’ end of the BCR sequence to use in analyzing sequences (Fig. 2B). We used hierarchical Bayesian mixture models in Rstan 2.21.8^70^ to bin sequences into cluster (“clones”). We assessed clonal diversity between groups using Hill Diversity Curves and Chao1 rarefication methods to account for individual variation in read depth, implemented using the Alakazam package (version 1.2.1) ^71–75^ (Fig. 2C). Statistical significance tests of the Hill Diversity Curves were done at the species level as described previously ^71^.*

Additionally, Lines 615-624: *We processed, filtered, and analyzed the sequencing BCR reads using pRESTO and previously described germline free identification methods^68,69^. We removed sequences with average Fastq quality score below 30. Consensus sequences were built from shared unique molecular identifiers (UMI). Sequences with low quality ends (<20) were trimmed. Sequences underwent sequential assembly. Both mouse and JFB reads which failed the R1 R2 alignment were aligned to mouse germline sequences and placeholder nucleotides “N” were filled into the R1-R2 gap. Sequences were only included in downstream analysis that IgBLAST classified as a functional BCR sequence (IgBlast version 1.18.0). For IgBlast, initially we used mouse germline V,D,&J sequences for both mouse and JFB sequences; however, no germline assignments were used in downstream analyses as we found a mismatch between the sequence and germline species introduced unpredictable biases and errors. Mouse germline sequences were obtained from the the international ImMunoGeneTics/GENE database (IMGT/GENE-DB) (CITE) on 2022-03-20. Finally, the 3’ C region of sequences were trimmed to the FWR4 region.*

Genome versions and citations (including the accession number) that were used for the BCR-seq processing should also be included.

R21: We now mention the IMGT database and the date it was accessed on lines 623-624.

Additionally, if not already included in the submission, the authors need to include a supplemental table with the BCR sequencing metrics for all samples:

at minimum this should include total reads sequenced

R23: Check

the number of mapped/aligned reads

R24: Check

the mapping/alignment rate

R25: Check

the number of reads used in the downstream analysis

R26: Check

This will also help a great deal with assessing the validity of their results

Thank you for the helpful suggestion, we have placed these information in in the text, lines 120-122.

Minor revisions:

There are some grammatical/spelling errors throughout the manuscript that need to addressed, including:
Line 52: "responses than were" should be "responses that were"

Done
        Line 72: "elicits" should be "elicit"

Done
Line 123: "germinal centers B cells" should be "germinal center B cells"

Done
Line 576: The FWR4 region is not part of the C region but is part of the V region located on the 5' end of the C region. This should be corrected in the last sentence.

Done **Figure 2: y-axis needs units, if it's fold change, then I recommend stating that in the axis label for clarity**

Thank you, the figure legend has been updated: “*Serum neutralization of Nipah-riVSV entry. Y axis units refer to the reciprocal of Nipah-riVSV entry, normalized to day 0*”
