# Supplementary material for: Bats generate lower affinity but higher diversity antibody responses than those of mice, but pathogen-binding capacity increases if protein is restricted in their diet
Source: PLoS Biol. 2024 Sep 24;22(9):e3002800. doi: 10.1371/journal.pbio.3002800 (PMC11421821; doi:10.1371/journal.pbio.3002800)
Supplement: Attachment — Submitted filename: Reviewer_responses_round_2.docx [file pbio.3002800.s010.docx]

a) During re-submission please indicate in the Financial Disclosure option the same statement you have within the manuscript.

We have updated the financial disclosure statement within the submission portal.

b) The Ethics statement should include the full name of the IACUC/ethics committee that reviewed and approved the animal care and use (which you have), as well as the protocol/permit/project license number (which is missing). Please include the specific protocol adhered to your ethics committee.

https://journals.plos.org/plosbiology/s/ethical-publishing-practice

Lines 332 to 335 now read: *Animal protocols were reviewed and approved by the MSU Institutional Animal Care and Use Committee (IACUC)* ***under protocol number 2021-174****. MSU is accredited by the Association for Assessment and Accreditation of Laboratory Animal Care (AAALAC; accreditation no. 713).*

c) You may be aware of the PLOS Data Policy, which requires that all data be made available without restriction: http://journals.plos.org/plosbiology/s/data-availability. For more information, please also see this editorial: http://dx.doi.org/10.1371/journal.pbio.1001797

Please supply the numerical values either in the a supplementary file or as a permanent DOI’d deposition for the following figures:

Figure 1BCEF, 2B, 3CDE, S1ABC, S2BC, S3B

NOTE: the numerical data provided should include all replicates AND the way in which the plotted mean and errors were derived (it should not present only the mean/average values).

The data and code to recreate all figures are available here: https://zenodo.org/records/12825679

The read file “all_code.R” allows a reader to recreate all figures and guides the reader through which data files go to which figures.

d) Please cite the location of the data clearly in all relevant main and supplementary Figure legends, e.g. “The data underlying this Figure can be found in S1 Data” or “The data underlying this Figure can be found in https://doi.org/10.5281/zenodo.XXXXX”

This is updated in the figure legends.

e) We require the original, uncropped and minimally adjusted images supporting all blot and gel results reported in the Figure 3B

We have the uncropped figure and will include this in the submission.

Thank you for your patience while we considered your revised manuscript "Bats generate lower affinity, but higher diversity antibody responses compared to mice, an effect that can be manipulated with diet" for consideration as a Short Reports at PLOS Biology. Your revised study has now been evaluated by the PLOS Biology editors, the Academic Editor and the original reviewers.

In light of the reviews, which you will find at the end of this email, we would like to invite you to revise the work to thoroughly address the reviewers' reports. As you can see below, the reviewers appreciate the effort made during the revision. However, after discussion with the Academic Editor, two issues need to be addressed before the study can be accepted for publication. In line with Reviewer #1, we suggest expanding the discussion on the specific risks of using inbred mice. This will provide a more compelling rationale for future experiments with outbred mice. Additionally, the technical issue highlighted by Reviewer #2 regarding sequencing depth must be addressed by following the reviewer's suggestions. While we think the manuscript is moving in the right direction and we and the reviewers are clearly interested in it, this technical concern is important and should be addressed.

R0: We have added more language and citations to why the use of outbred mouse strains in comparative immunology studies would be beneficial moving forward. Lines 293 to 302 now read: *Furthermore, as we begin to manipulate the diet and housing conditions of bats, we also need to consider the housing conditions, and strains, of mice. In our study, we used the inbred BALB/c mouse strain. However, incorporating outbred mouse strains could better match the genetic diversity found in our study’s bats. Mouse inbreeding has led to divergences in the VDJ genes between the commonly used BALB/c and C57BL/6 strains^59^, a loss of germline diversity in the BALB/c light chain region ^60,61^, and changes to the B cell response to immunization ^62^. We should also consider using pathogen naturalized mice (mice which have encountered various pathogens in their environment) in future studies ^reviewed in^ ^46^. These mice have different circulating lymphocyte populations and it would be useful to assess if mice with prior microbial exposure changes their B-cell receptor diversity following immunization. The incorporation of naturalized and outbred mice in future comparative immunology studies involving bats, wild or captive, could yield more relevant and insightful results.*

Reviewer #1:

Thank you for carefully addressing the points I raised in my comments. The manuscript now holds together much better. While I'm not fully convinced that all the pieces fit seamlessly, I believe the rationale and conclusions drawn from the data are sufficiently argued.

----------------------------------------------------------------

Reviewer #2:

The authors have done a great job of responding to the numerous reviewer comments. Let me say first that I appreciate how Herculean this effort is and the challenge of assessing humoral immunity in non-model species. In particular, I think the reframing of the manuscript, removal of the pilot, and the addition of the information about where samples were not available makes the story much clearer and cleaner. I also really appreciate the author's transparency on their methods including the submission of their R script assessing read depth. This was incredibly informative for my understanding of their BCR sequencing methods. Having clarified this I have one outstanding concern that I think is required for understanding their results and necessitates a reanalysis and several more minor suggestions to strengthen the clarity of the manuscript. I think this is a really interesting piece of work and am excited for it to contribute to our knowledge but would like to ensure that the results are as accurate as possible given experimental constraints and that they are not over interpreted.

Major concern: Thank you so much for including the R code you used to test the sensitivity of Alakazam to read depth. I now can better articulate my concern. In figure 3c it is evident that your sequencing depth, especially for mesenteric lymph nodes, but also for the spleens is very, very low. A MiSeq can theoretically produce 44-50 million reads passing filter -- a back of the envelope calculation using your highest read count (~2000) for every individual and sample is 0.3% of the sequencing capacity of a MiSeq run even if you grouped all the samples on a single run. Especially for splenic samples, I'd expect at least tens of thousands of reads if not more. This is very very low and makes me wonder about what went wrong in the library preparation and/or sequencing compared to the NP-Ficoll and NP-CGG experiments when the same protocol was used. I do not want to further delay the publication of this work as I know the authors have worked long and hard on this manuscript,

however, if there is remaining amplicon library/ cDNA, etc. it would be very valuable to remake libraries and/or resequence to increase sampling depth. I think this is the biggest challenge of making inferences from this data because the data are inherently biased for assessment of diversity.

R1) Thank you for pointing this out. During the BCR amplification step, we add on a unique molecular identifier (UMI) to each cDNA strand. This UMI gets attached to the cDNA during the RT step and therefore we can track errors introduced to a single read by the amplification steps. The UMI is a set of 14 random base pairs. During the cleaning step, we collapse reads which share a UMI down to a single consensus read.

We ran the experiments with different miseq barcodes. We obtained the read counts from one of our miseq run that contained sequences from our BCR sequences. Pre cleaning, we had comparable read counts for three barcodes: #GCCAAT: 1,869,479,#ACAGTG: 1,968,730, & #CAGATC: 2,182,838. CAGATC contained the food restriction experiment counts. Looking at one set of reads, when we collapse the sequences down to consensus reads (using the UMI) we end up with the following counts: ACAGTG: 762,788, GCCAAT: 484,787, CAGATC: 77,455.

We are losing a disproportionate number of reads for the food restriction barcode at this step (and only this step). **However**, after this step we also have a ~7x higher ‘consensus count’ for sequences in CAGATC. Our interpretation is 1) the miseq library prep and sequencing went as expected, but 2) we had few BCR mRNA molecules in the original tissues from bats in the food restriction experiment. When we start the BCR library prep we have comparable RNA ug amounts that go into the 5’ RACE reaction from all animals. However, after running the 5’ RACE RT and PCR steps we had far less micrograms of DNA in the food restriction experiment. This was evidenced by qubit and the western blots were fainter for the target amplicon. This made sense to us as those bats in the food restriction experiment were not boosted and did not make a particularly strong antibody response.

We are updating the section in the paper where we describe sequence counts more thoroughly. Lines 122 through 124 will now read:

*During the library preparation, a unique molecular identifier (UMI) was added to each cDNA. During sequence cleaning, reads that shared a UMI were collapsed into a consensus read. Prior to building a consensus sequence, we had approximately 180,000 reads per sample from the bat and mouse comparison study and 90,000 reads per sample from the food restriction study. After collapsing reads, we had approximately 59,000 reads per individual in the mouse and bat comparison study, but only 3,600 reads per individual in the food restriction study and the average consensus count per sequence was approximately 7x higher. Of those reads, 61% had both a V, D, & J segment that was identifiable by IgBlast and was used in downstream analyses.*

For reference, the alignment free method you cite for your BCR identification was tested on roughly 30K samples per distribution and was tested on datasets of equal size (aka "sampling depth"). It is here you need to subsample to the lowest number of reads -- in the clone identification -- not alakazam -- to determine whether your BCR diversity findings are accurate. You are feeding clone data from this initial assessment into alakazam and so the underlying distributions of clone calls are already biased. I think this is why the diversity estimates are so strongly (at least visually) correlated with the read counts. I bet if you plotted each individuals calculated diversity metric against its read depth there'd be a strong correlation. At minimum, I would like to see the impact of subsampling at the alignment free step -- e.g. subsampling your individual sequences to whatever the lowest sequence number is before sticking it in the python script and then doing the Hills
numbers in R -- and a graph of read depth against diversity.

R2) Thank you, this is a thoughtful suggestion. We have now run a test where we processed the data from the food restriction experiment four ways. In all four steps we subsampled to the smallest individual read count prior to establishing a clonal cutoff (per your suggestion). We set the smallest read count based on either raw sequencing reads or the collapsed reads using on the unique molecular identifier. We then used the germline free method or the Immcantation’s Shazam’s (<https://shazam.readthedocs.io/en/stable/vignettes/DistToNearest-Vignette/>) method which uses a mouse germline and a hamming distance to assign clones. Since this comparison only included bats (no species comparison) and is not for publication we thought this would be okay in this instance.

Under three of the four settings, we still arrive at the same conclusion. However, under one setting we do not see any differences between the diet groups.

Overall, for the food restriction experiment, we are confident there were real differences between the B cell response by tissue and diet condition. However, your suggestions have made us question if the B cell diversity metrics are the best and more accurate way of representing those differences and to what extent they are a product of read depth. We want to use cautious language here and so we tempered our conclusions and do not emphasize this finding in the conclusion section:

Lines 197 to 200 have been slightly modified : *However, Hill diversity curves with Chao1 rarefication may have unreliable estimates when sample sizes are low or uneven^42,43^, which is what we observed in the food restriction experiment, especially from mesenteric lymph nodes harvested from bats on the fruit only diet (Fig. 3C). Thus, these Hill diversity estimates should be interpreted with caution and could be driven primarily by read depth.*

Minor comments:

Lines 90-116 and Figure 1: It's odd to me that you refer to parts of figure 1 in the text out of order A then D then B then C. I don't know if there's a way to rearrange the figure to flow with the text but it might be worth thinking about. Similarly in Figure 1 E you discuss IgM first in the text so maybe put that on top?

R3) Thank you. That is another helpful suggestion, our objective with the figure was to lay it out as a grid with NP-CGG and NP-Ficoll aligned vertically. We have tried to rearrange the figure, but we are struggling to do so while maintaining this grid.

We can easily rearrange the IgM and IgG, however.

Also in Figure 1E, it's hard to see the overlap in the IgG NP-Ficoll graph. Given that the y axes are not the same in each graph I'd just zoom in so it's clearer and there's less unnecessary white space.

R4) Thank you, we have changed the axes of these plots and agree it makes the differences clearer (see R3).

Figure 1F: I think the mouse and bat colors are switched. (Text reports the opposite findings...)

R5) Thank you, it is the CT value we are reporting (so higher is lower expression), and we will change the figure to make that clearer.

Lines 113-115: You say that mice couldn't displace the B1-8 antibody on day 56 but in your figure there's a significant difference.

R6) Thank you for catching this and reading the manuscript so carefully. The text on lines 130 to 133 now reads: “*Sera from NP-Ficoll (T.I.) immunized bats and mice could not displace the B1-8 antibody on days 21 nor day 35, indicating SHM and affinity maturation are required to generate antibodies which compete with the B1-8 antibody (Fig. 1C). Mouse from NP-Ficoll (T.I.) immunized mice could displace the competitor antibody on day 57.*”

Line 213: Be careful with how you're using "clonal diversity". In line 208 you use "high clonal diversity" and in 213 you use "low clonal diversity" to describe the same phenomenon. My guess is in 213 you want to use "fewer dominant clones".

R7) We agree that phrasing is more precise. Lines 246 to 249 now say: *If bat germinal centers are more permissive, enabling low affinity B cells to continue proliferating, or if plasma cell differentiation rates are lower in bats, this could result in the fewer dominant B cell clones and contribute to low avidity serum antibodies.*

230-231: The two uses of "counterintuitive" is a little awkward. I'd remove the introductory phrase.

R8) We agree. Lines 265-268 now read*: Specifically, restricting their dietary protein increased serum antibody levels to both a replication incompetent Nipah-G VSV and a live virus, H18N11 IAV. Similar findings to our study have been observed in wild animals, where, counterintuitively, improved access to food has been associated with decreased antibody levels^25,28–32^.*

Other suggestions:
I saw in the reviewer comments your note about the lymph nodes. I think this is a great contribution to the field about how to find them, etc. and I think it'd be a great note for an anatomy journal or the like!
